# Phosphoproteome dynamics mediate revival of bacterial spores

**DOI:** 10.1186/s12915-015-0184-7

**Published:** 2015-09-17

**Authors:** Alex Rosenberg, Boumediene Soufi, Vaishnavi Ravikumar, Nelson C. Soares, Karsten Krug, Yoav Smith, Boris Macek, Sigal Ben-Yehuda

**Affiliations:** Department of Microbiology and Molecular Genetics, Institute for Medical Research Israel-Canada (IMRIC), The Hebrew University-Hadassah Medical School, The Hebrew University of Jerusalem, POB 12272, 91120 Jerusalem, Israel; Proteome Center Tuebingen, Interfaculty Institute for Cell Biology, University of Tuebingen, Auf der Morgenstelle 15, 72076 Tuebingen, Germany; Genomic Data Analysis Unit, The Hebrew University - Hadassah Medical School, The Hebrew University of Jerusalem, 91120 Jerusalem, Israel

**Keywords:** *Bacillus subtilis*, Germination, Phosphoproteome, Spore

## Abstract

**Background:**

Bacterial spores can remain dormant for decades, yet harbor the exceptional capacity to rapidly resume metabolic activity and recommence life. Although germinants and their corresponding receptors have been known for more than 30 years, the molecular events underlying this remarkable cellular transition from dormancy to full metabolic activity are only partially defined.

**Results:**

Here, we examined whether protein phospho-modifications occur during germination, the first step of exiting dormancy, thereby facilitating spore revival. Utilizing *Bacillus subtilis* as a model organism, we performed phosphoproteomic analysis to define the Ser/Thr/Tyr phosphoproteome of a reviving spore. The phosphoproteome was found to chiefly comprise newly identified phosphorylation sites located within proteins involved in basic biological functions, such as transcription, translation, carbon metabolism, and spore-specific determinants. Quantitative comparison of dormant and germinating spore phosphoproteomes revealed phosphorylation dynamics, indicating that phospho-modifications could modulate protein activity during this cellular transition. Furthermore, by mutating select phosphorylation sites located within proteins representative of key biological processes, we established a functional connection between phosphorylation and the progression of spore revival.

**Conclusions:**

Herein, we provide, for the first time, a phosphoproteomic view of a germinating bacterial spore. We further show that the spore phosphoproteome is dynamic and present evidence that phosphorylation events play an integral role in facilitating spore revival.

**Electronic supplementary material:**

The online version of this article (doi:10.1186/s12915-015-0184-7) contains supplementary material, which is available to authorized users.

## Background

In response to nutrient deprivation, bacteria of various *Bacillus* species can carry out a complex developmental process called sporulation, resulting in the formation of a highly durable spore, the most resilient cell type known. Consequently, spore forming bacteria, including dangerous pathogens, such as *Clostridium difficile* (*C. difficile*) and *Bacillus anthracis* (*B. anthracis*), are highly resistant to antibacterial treatments and difficult to eradicate [1–4]. Bacterial spores survive for long periods of time and can endure extremes of heat, radiation, and chemical assault including antibiotics. Remarkably, once conditions become favorable, the spore can rapidly revive and convert into an actively growing cell [1].

The robustness of the spore is facilitated by its unique structure and chemical composition. The spore core, containing DNA, RNA, and protein components, exhibits distinctive physiological conditions such as low pH (6.0 ± 0.3) and reduced water content, alongside a high concentration of pyridine-2,6-dicarboxylic acid (dipicolinic acid; DPA) [5, 6]. The core is protected from the environment by a relatively impermeable inner membrane, which in turn is encased by a thick peptidoglycan layer, called a cortex. The cortex is further shielded by multiple layers of a proteinaceous coat, comprising more than 70 proteins (Cot proteins) synthesized during sporulation [4, 7, 8]. The chromosome, the most vital molecular constituent within the spore core, is protected by specialized spore-specific DNA binding proteins, known as the α/β-type small acid-soluble spore (Ssp) proteins [9]. Ssp proteins bind DNA in a non-specific manner condensing it into a ring-like structure, thereby changing its conformation from an active to an inactive form [10]. Ssp binding also alters the DNA photochemical reactivity upon UV exposure, leading to the formation of spore-specific photoproducts instead of thymine dimers [11]. These global modifications facilitate the protection of the spore DNA from UV, γ-radiation, and heat during the dormant state and while exiting dormancy [12, 13].

**Fig. 1:**
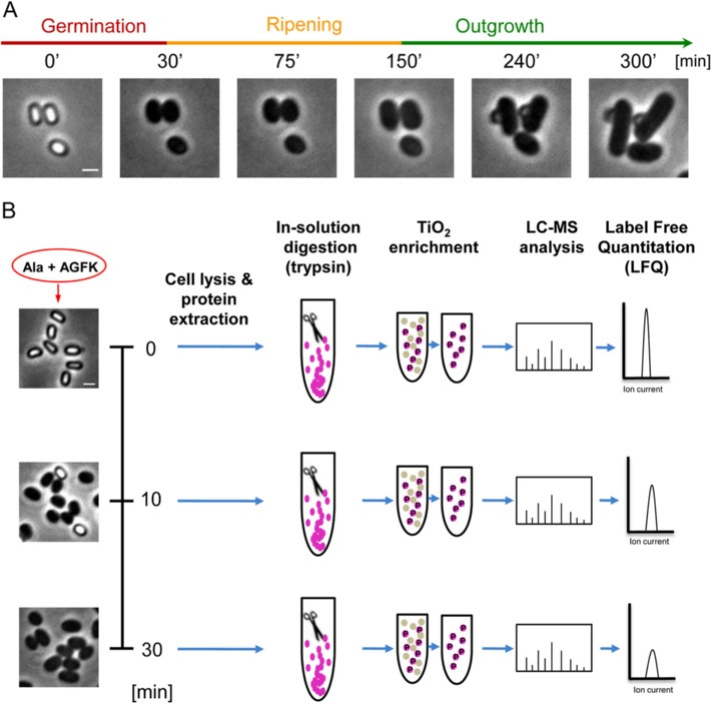
The proteomics workflow utilized for defining the germinating spore phosphoproteome. **a** The sequence of events during spore revival of wild-type *B. subtilis* (PY79) as captured by phase-contrast microscopy. Shown are phase-contrast images acquired at the indicated time points. Scale bar represents 1 μm. **b** Purified spores of PY79 strain were incubated in a revival medium containing L-Ala and AGFK, and were harvested at the indicated time points. Left panels show corresponding phase-contrast images of the germinating spores prior to further processing. Next, spores were lysed, and proteins were extracted and subjected to trypsin digestion. Digested peptides were enriched for phosphorylated peptides by TiO_2_ chromatography (five consecutive rounds). All samples were analyzed on LTQ-Orbitrap XL mass spectrometer. Label-free quantitation (LFQ) was applied to determine phosphoproteome dynamics (see Methods)

During dormancy, the spore remains responsive to its environment and commences revival upon sensing various stimuli, such as DPA, nutrients (L-amino acids, D-sugars, and purine nucleosides), or muropeptides [8, 14]. The revival process has been classically divided into two major consecutive phases: (1) germination, in which nutrients bind to germinant receptors located in the spore inner membrane, triggering the release of monovalent cations (H^+^, K^+^) and DPA, spore rehydration, cortex hydrolysis, and coat disassembly; we have recently shown that the completion of this process requires active translation [15]. During germination, the optical properties of the spore are altered, resulting in a conversion from a phase-bright spore to a phase-dark cell (Fig. 1a). (2) Outgrowth, in which a new cell starts to emerge from the disintegrated coat, followed by conversion into a vegetative cell (Fig. 1a) [16]. Previously, we identified an intermediate phase, designated the “ripening period”, taking place after germination prior to cell outgrowth. The ripening period is exploited by the germinating spore for molecular reorganization, while no morphological changes are evident (Fig. 1a). Key events that occur during ripening include the syntheses of rRNA and ribosomal proteins, the core RNA polymerase components, and energy-producing enzymes along with degradation of spore-specific proteins [15, 17]. During this process, mutations acquired in the dormant state are repaired, thus enabling the spore to commence DNA replication [18, 19]. Here, we will refer to the entire process of spore growth resumption, which includes germination, ripening, and outgrowth, as “spore revival”.

Nutrient binding to germinant receptors leads to rapid recommencement of metabolism, transcription, and translation [15, 16, 20–22]; however, the molecular mechanism propelling these dramatic changes remains largely unknown. A possible mechanism for co-initiation of multiple cellular processes could be through post-translational modifications such as protein phosphorylation, which is exploited by bacteria to mediate rapid cellular responses to various external stimuli [23]. In line with this view, a selection of protein kinase inhibitors were shown to perturb the process of germ tube formation upon exiting dormancy in the bacterium *Streptomyces coelicolor* [24]. Further, it has been shown that muropeptides trigger spore germination in *B. subtilis* by activating the eukaryotic-like membrane serine/threonine kinase PrkC. In turn, PrkC phosphorylates the essential translational initiation component elongation factor G (EF-G). Yet, the consequences of this phosphorylation event and its effect on germination are not fully understood [14].

Here, we examined if and to which extent protein phosphorylation occurs at the onset of spore revival. By utilizing high resolution mass spectrometry (MS)-based phosphoproteomics, we defined the reviving *Bacillus subtilis* (*B. subtilis*) spore Ser/Thr/Tyr phosphoproteome, and provided evidence that widespread dynamic changes indeed take place upon induction of germination. The uncovered phosphoproteome of the germinating spore contained proteins belonging to central biological processes, including translation, transcription, carbon metabolism, and spore-specific constituents. Subsequently, by altering phosphorylation sites in key factors facilitating these processes, we revealed the crucial effect phosphorylation plays in driving the exit from dormancy.

## Results

### The phosphoproteome of a reviving spore is dynamic and spans key biological processes

To explore whether spore revival involves phosphorylation events in spore proteins, we sought to characterize the phosphoproteome of a germinating spore. To this end, *B. subtilis* spores were purified and induced to germinate in buffer supplemented solely with the defined germinants L-Ala and AGFK (asparagine, glucose, fructose, and potassium) [25]. We reasoned that a combination of germinants would efficiently trigger the signaling pathways, activating synchronous revival. Importantly, such conditions allow spores to complete germination, but not outgrowth, leaving them trapped at the early ripening period [15, 26]. The germinating spores were sampled in three biological replicates at different time points: t = 0 minutes, at the dormant state; 10 minutes post-germinant addition, when the majority of the spores (80 %) had completed germination; and subsequently at 30 minutes, when all spores had germinated and yet were arrested at early ripening (Fig. 1b, left panels). Spores from each time point were lysed, and their protein content extracted, trypsinized, and enriched for phosphopeptides prior to MS analysis (Fig. 1b).

**Fig. 2:**
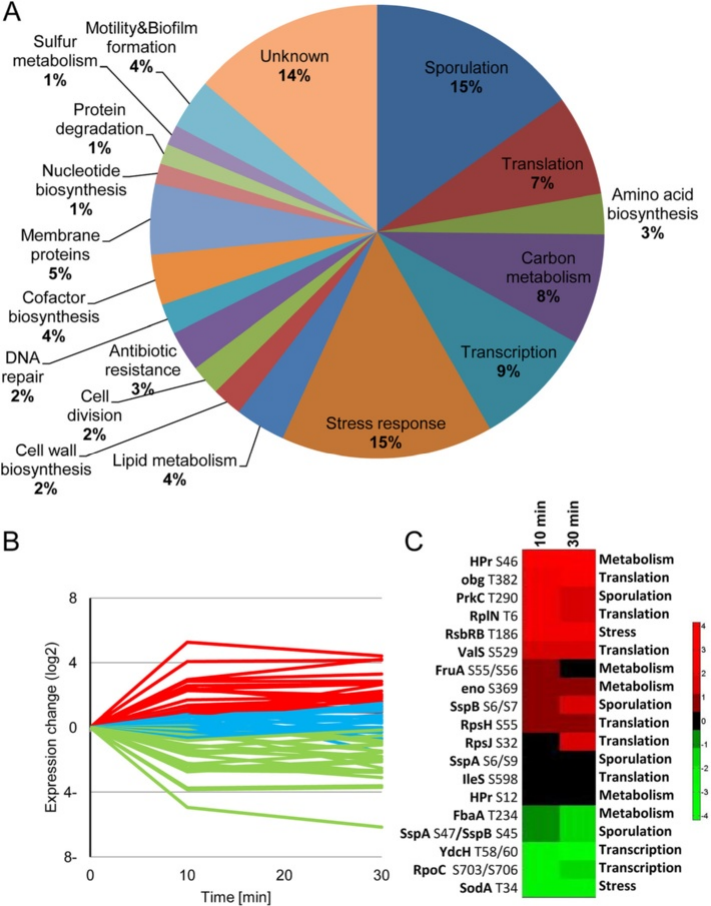
The reviving spore phosphoproteome. **a** The uncovered phosphoproteome of the germinating *B. subtilis* spore spans proteins involved in the indicated biological processes, as assigned by the DAVID functional annotation tool. Data was extracted from (Additional file 2: Table S1). **b** Phosphorylation profiles of sites detected across 0, 10, and 30 minutes of spore germination. Green – decrease in phosphorylation levels, Red – increase in phosphorylation levels, Blue – no significant change in phosphorylation levels. Data was extracted from Additional file 4: Table S2. **c** Hierarchical clustering analysis of the indicated phospho-site amino acids comparing 0 to 10- and 30-minute time points. Listed sites belong to proteins representative of central biological processes as defined in (**a**)

As a first step in our investigation, we defined the phosphoproteome of a germinating bacterial spore by combining data from all analyzed replicates and time points. Peptide and protein identification was performed by MaxQuant (Andromeda search engine) using a target-decoy strategy, as described previously [27, 28]. The false discovery rate at the protein level was 1.7 % and at the peptide level 0.4 %; in all cases, protein and peptide intensities showed a high level of correlation between technical replicates (Pearson correlation coefficient >0.95; Additional file 1: Figure S1). Collectively, our analysis revealed a total of 155 phosphorylation sites located in 124 different phosphoproteins (Additional file 2: Table S1) detected at all time points for a given experiment. The majority of the phosphorylation events (125 sites within 106 proteins) had not been detected in previous phosphoproteome analyses of vegetative *B. subtilis* cells (Additional file 2: Table S1) [29–35]. Phosphorylation events were found to occur mainly on Ser/Thr sites (120/155), with a minority (35/155) located on Tyr residues (Additional file 2: Table S1), similarly to the distribution observed in exponentially growing *B. subtilis* cells [29, 30, 33, 34]. The uncovered phosphoproteome of the germinating spore spans proteins involved in key biological functions, including translation, transcription, carbon metabolism, stress response, and spore-specific determinants (Fig. 2a; Additional file 2: Table S1).

To further investigate the difference between the vegetative and spore phosphoproteomes, we compared the spore germination results with our recent study, in which we detected 177 phosphorylation sites on 140 *B. subtilis* proteins and quantified their dynamics during vegetative growth [30]. Of 140 phosphoproteins previously monitored, only 28 were detected as phosphorylated during spore germination. Moreover, of 42 phosphorylation sites on these proteins, only 17 were detected in the spore germination dataset, confirming existence of a unique spore phosphoproteome. Intriguingly, in vegetative cells, a majority of the 17 common phosphorylation sites were peaking in late stationary phase, pointing at their possible involvement in processes typical for this stage, including entry into sporulation (Additional file 3: Figure S2 and Additional file 4: Table S2).

Next, we assessed whether germination induces multiple changes in protein phosphorylation state. To explore this possibility, differences in phosphorylation levels between the dormant stage (t = 0) and the consecutive 10 and 30 minute time points were determined by label free quantitation (LFQ) utilizing the results from one of the three biological replicates, which was measured in technical triplicates and resulted in the most comprehensive data set (see Methods and Additional file 5: Figure S3A and S3B). Of note, the remaining two biological repeats had a relatively low coverage. The reason for that was partly biological, due to low occupancy of most of the phosphorylation events, and partly technical, due to the high background of unmodified peptides even after phospho-enrichment.

Utilizing this approach, we succeeded in monitoring phosphorylation dynamics at 74 localized phospho-sites belonging to 58 different proteins (Additional file 6: Table S3). Phosphorylation site intensities were normalized to the overall level of their respective proteins to eliminate possible bias due to changes in total protein abundance. Statistical analysis of quantification data was performed by calculation of “Significance B” value (*P* = 0.05) of ratios of LFQ intensities at the protein level (Additional file 5: Figure S3C and S3D). This analysis showed that most of significantly changing proteins had log_2_ ratios −2 < x > +2, and therefore these values were used as an arbitrary cutoff for significance of regulation. Remarkably, the phosphorylation level at the majority of sites increased or decreased considerably already 10 minutes post-induction of germination. The observed trend for a given site generally persisted or became more pronounced at the 30 minute time point (Fig. 2b; Additional file 6: Table S3). Temporal changes in phosphorylation levels were abundant in proteins involved in spore-specific functions, translation, metabolism, and stress response (Fig. 2c; Additional file 6: Table S3). In addition, we followed the recently published procedure of Sharma et al. [36] to estimate occupancies of phosphorylation sites from LFQ data. Due to stringent acceptance criteria for such calculation (existence of singly phosphorylated, modified and unmodified counterpart peptides and unmodified protein ratio), we could estimate the occupancies of only 13 phosphorylation sites. Nevertheless, the median estimated occupancy was relatively high (69 %), confirming increased phosphorylation levels on at least 10 proteins (Additional file 7: Table S4). Combined, these results indicate that phosphorylation events are extensive during germination, and could play a crucial role in fueling the revival process by activating key cellular pathways.

### A strategy for assessing the significance of phosphorylation dynamics

We next attempted to explore the biological significance of the identified phosphorylation events on the progression of spore revival. To this end, we chose to investigate representative proteins of key functional groups that possess phospho-sites highly conserved among *Bacillus* species and display a dynamic phosphorylation pattern during germination. Currently, amino-acid substitution is the most feasible way to conduct a large scale analysis of phospho-modifications [37]. Hence, the selected proteins were mutated to either abolish phosphorylation potential by replacing Ser/Thr/Tyr with Ala, or mimic a constitutive phosphorylation state by replacing Ser/Thr/Tyr with Asp. Mutant alleles, encoding the modified proteins, were constructed by site-directed mutagenesis and introduced into their native sites within the *B. subtilis* genome as the sole chromosomal copy using a “pop in pop out” strategy (see Methods) [38]. Mutant alleles encoding for proteins involved in the following processes were generated: (1) spore-specific proteins that need to be removed during revival (i.e. SspA, SspB), (2) components of the translational machinery required to be activated early on (i.e. ribosomal protein S10 (RpsJ), EF-G, EF-TU), and (3) carbon utilization factors that are necessary for an immediate initiation of energy production (i.e. histidine-containing phosphocarrier protein (HPr), encoded by *ptsH*). We cannot exclude the possibility that mutating phospho-site amino acids might affect protein function for reasons other than phosphorylation state. Therefore, the consequence of the generated phosphorylation mutants was compared to that of a corresponding null mutant when possible. Of note, additional proteins were initially examined but excluded from further analysis, as deletion and/or phospho-site-specific mutations of their encoding genes had no obvious effect on revival (Additional file 8: Table S5). All the revival experiments presented below were done using L-Ala as a germinant; however, similar results were obtained when AGFK was used as a germinant (data not shown).

### Newly identified phosphorylation sites of α/β-type Ssp proteins affect spore revival

A substantial group of proteins that exhibited dynamic phospho-modifications was comprised of spore-specific components. Notably, multiple phosphorylation sites were detected on the major Ssp proteins SspA, SspB, and SspE (Fig. 2c; Additional file 2: Table S1 and Additional file 6: Table S3). To date, such post-translational modifications have not been reported for Ssps. To investigate the effect of Ssp protein phosphorylation on spore revival, we focused on SspA, the most abundant α/β-type Ssp [39]. A total of four phospho-sites were identified within SspA: Ser6 and Ser9 at the N-terminus, Ser47 in the second alpha helix that interacts with the DNA, and Ser58 located in the C-terminus (Additional file 2: Table S1 and Additional file 9: Figure S4) [40]. Phosphorylation dynamics on Ser6, Ser9, and Ser47 residues were successfully quantified, with the N-terminal residues (Ser6 and Ser9) exhibiting relatively constant phosphorylation levels, while the alpha helical site Ser47 displaying a strong dephosphorylation trend post-initiation of revival (Fig. 2c; Additional file 6: Table S3). Notably, Ser47 site is also completely conserved among *Bacillus* species (Additional file 9: Figure S4A) and between the SspA and SspB proteins; therefore, we first investigated the consequences of mutating this phospho-site.

**Fig. 3:**
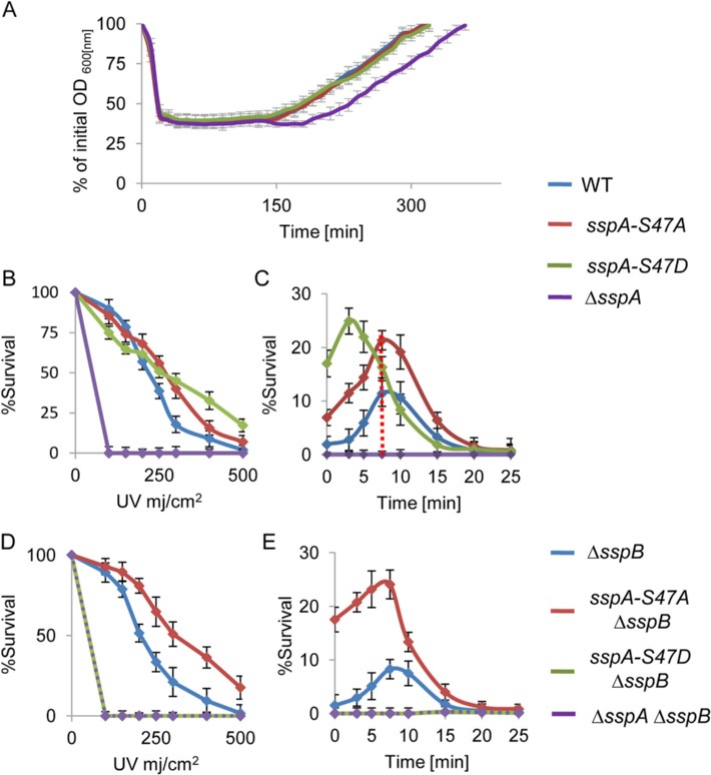
Characterization of SspA-S47 phosphorylation-site mutants. **a** Spores of PY79 (wild type, WT), AR209 (*sspA-S47A*), AR210 (*sspA-S47D*), and AR179 (∆*sspA*) strains were incubated at 37 °C in S7-defined medium supplemented with L-Ala (10 mM), and optical density (OD_600_) was measured at the indicated time points. Data are presented as a fraction of the initial OD_600_ of the phase-bright spores. Decreasing OD_600_ signifies spore germination while increasing OD_600_ indicates spore outgrowth. The data points are averages of results obtained from four independent biological repeats. Error bars designate SD. **b** Spores of PY79 (wild type, WT), AR209 (*sspA-S47A*), AR210 (*sspA-S47D*), and AR179 (∆*sspA*) strains were exposed to increasing UV (254 nm) doses (mj/cm^2^) and plated on LB for viable count. Survival was calculated by dividing the viable spore titer at any given UV dose (mj/cm^2^) with the spore titer obtained from the non-irradiated spores. The data points are averages of results obtained from three independent biological repeats. Error bars designate SD. **c** Spores of PY79 (wild type, WT), AR209 (*sspA-S47A*), AR210 (*sspA-S47D*), and AR179 (∆*sspA*) strains were germinated with L-Ala (10 mM). Samples were taken at the indicated time points, irradiated with 500 mj/cm^2^ UV (254 nm), and plated on LB. Survival was calculated by dividing the viable spore titer at any given time point with the spore titer obtained from spores irradiated at the 0 time point. The data points are averages of results obtained from three independent biological repeats. Error bars designate SD. **d** UV resistance of AR186 (∆*sspB*), AR195 (∆*sspA* ∆*sspB*), AR187 (*sspA-S47A *∆*sspB*), and AR188 (*sspA-S47D *∆*sspB*) spores was determined as described in (**b**). **e** UV resistance of AR186 (∆*sspB*), AR187 (*sspA-S47A*, ∆*sspB*), and AR188 (*sspA-S47D*, ∆*sspB*) germinating spores was determined as described in (**c**)

Strains producing SspA-S47A or SspA-S47D were constructed and their ability to sporulate and revive was assessed. The mutant strains sporulated with efficiency comparable to wild type, and their revival capability, as measured by monitoring changes in optical density (OD_600_), was not affected, while ∆*sspA* spores exhibited an extended ripening period (Fig. 3a; Additional file 10: Table S6). Since a major function of SspA is protecting the spore DNA from UV damage, we assayed the UV resistance of the mutant strains. Irradiating spores harboring the mutant alleles revealed slightly modified survival kinetics in comparison to wild type spores, while ∆*sspA* spores were highly sensitive to UV under the tested conditions (Fig. 3b). Additionally, an altered profile of resistance was observed when mutant spores were irradiated during germination (Fig. 3c). These results suggest that the phosphorylation state of Ser47 affects the protein functionality.

**Fig. 4:**
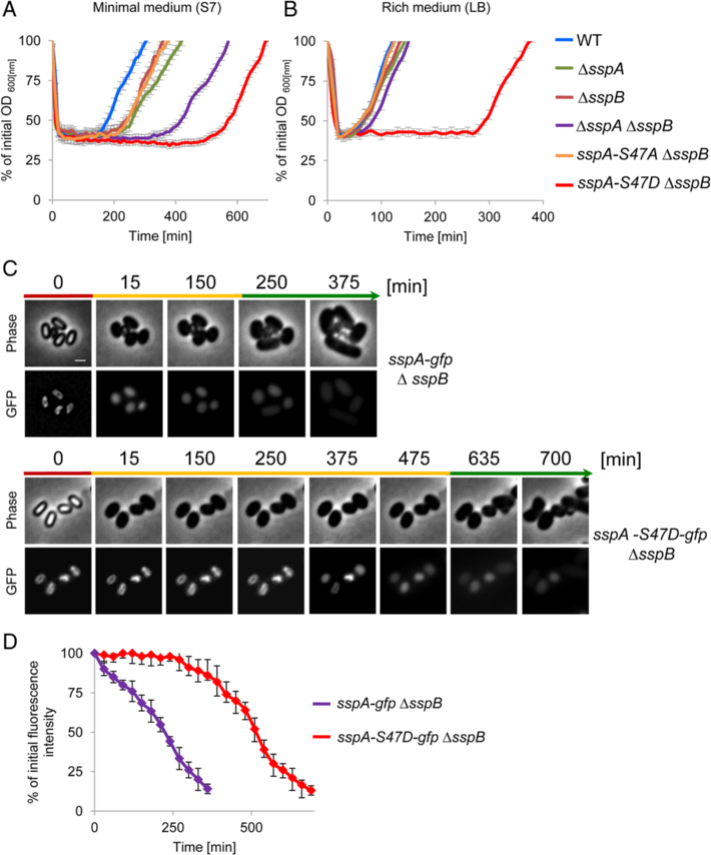
SspA-S47D ∆*sspB* imposes delay on spore revival. (**a**, **b**) Spores of PY79 (wild type, WT), AR186 (∆*sspB*), AR179 (∆*sspA*), AR195 (∆*sspA* ∆*sspB*), AR187 (*sspA-S47A* ∆*sspB*), and AR188 (*sspA-S47D* ∆*sspB*) were incubated at 37 °C in S7-defined medium (**a**) or LB (**b**) supplemented with L-Ala (10 mM) and optical density (OD_600_) was measured at the indicated time points. Data are presented as a fraction of the initial OD_600_ of the phase-bright spores. Decreasing OD_600_ signifies spore germination while increasing OD_600_ indicates spore outgrowth. The data points are averages of results obtained from three independent biological repeats. Error bars designate SD. **c** Spores of AR200 (*sspA-gfp*_*A206K*_ ∆*sspB*) and AR206 (*sspA-S47D-gfp*_*A206K*_ ∆*sspB*) were incubated in S7-defined medium supplemented with L-Ala (10 mM) and monitored by time lapse microscopy. Shown are phase-contrast (upper) and fluorescent (lower) images taken at the indicated time points. Arrows signify the dominating revival stages of each population at a given time point (germination, red; ripening, orange; elongation, green). Shown is a representative experiment out of three independent biological repeats. **d** Quantification analysis of the level of the GFP signal measured from the spores in (**c**) at the indicated time points, calculated as percent of the initial intensity at t = 0. For each time point, the calculated net average fluorescence intensity from at least three different fields (spores n >300) was averaged, and the intensity of wild type spores (PY79), lacking *gfp*, was subtracted. Error bars designate SD

SspB, which also underwent phosphorylation on its alpha helical site Ser45 (equivalent to SspA-Ser47 position) and the N-terminus (Fig. 2c; Additional file 2: Table S1 and Additional file 6: Table S3), constitutes, together with SspA, approximately 80 % of the *α*/*β*-type Ssp pool [9]. Since SspA and SspB have been demonstrated to possess overlapping functions [39], we reasoned that SspB might compensate and obscure the phenotypes caused by the point mutations at the SspA-Ser47 phospho-site. Therefore, we repeated our analysis of *sspA-S47* mutant alleles using strains harboring a deletion for *sspB.* Indeed, the absence of SspB uncovered a distinct phenotype for the SspA-S47D phospho-mimetic mutation. The *sspA-S47D* ∆*sspB* strain was perturbed in sporulation, producing spores with a substantial defect in UV resistance in comparison to ∆*sspB* spores (Fig. 3d; Additional file 10: Table S6). Consistent with this finding, *sspA-S47D* ∆*sspB* spores irradiated during germination failed to survive, similarly to the ∆*sspA* ∆*sspB* mutant spores (Fig. 3e). Importantly, in the absence of *sspB*, spores bearing the *sspA-S47A* allele, which abolishes phosphorylation potential, exhibited opposing phenotypes. This strain showed an increased UV resistance during dormancy and germination (Fig. 3d, e). Furthermore, *sspA-S47D* ∆*sspB* spores exhibited a markedly extended ripening period (300 min), which was longer than that of the ∆*sspA* ∆*sspB* mutant spores (200 min; Fig. 4a). Ssp proteins have been shown to serve as an amino acid reservoir for spores reviving under nutrient-limiting conditions [41], such as those employed in our study. To rule out the possibility that the extended ripening period displayed by *sspA-S47D* ∆*sspB* spores is due to lack of nutrients, spores were induced to revive in rich LB medium. Under these conditions, only *sspA-S47D* ∆*sspB* spores maintained a pronounced extension of the ripening period (Fig. 4b), implying that the mutant protein has an altered functionality.

Reduced UV resistance and delayed outgrowth were reported to be features of spores produced by Ssp mutants with elevated affinity to DNA [42, 43]. To further explore if the *sspA-S47D* ∆*sspB* observed phenotypes are of a similar nature, we followed the localization of SspA-S47D-GFP in the absence of SspB. In dormant spores, a typical ring-like structure similar to that of the SspA-GFP was observed (Fig. 4c), indicative of the mutant ability to bind DNA. During revival, SspA-GFP ring was rapidly replaced by a diffuse pattern with a subsequent decrease of the protein signal (Fig. 4c, d). On the other hand, SspA-S47D-GFP maintained the ring-like structure for approximately 400 min prior to the transition into the diffuse pattern (Fig. 4c, d).

Since SspB harbors a homologous SspA-Ser47 site (SspB-Ser45), we next investigated the consequences of mutating this position in WT and SspA null backgrounds. The resulting phenotypes were similar to those observed for SspA-S47A/D, namely altered UV sensitivity and extended ripening period (Additional file 11: Figure S5). Moreover, a strain harboring both SspA-Ser47D and SspB-Ser45D showed high UV sensitivity and prolonged revival dynamics (Additional file 11: Figure S5). It is therefore conceivable that phosphorylation of these sites affects Ssp-DNA interaction, with the investigated mutants having higher affinity to DNA than the wild type.

We also constructed additional Ssp mutant strains by modifying the phosphorylation sites located at the N- and C-terminal parts of SspA and SspB, i.e. SspA-S6A,S9A,S58A, SspA-S6D,S9D,S58D, SspB-S6AS7A, and SspB-S6D,S7D. The key experiments described above were conducted in the presence and absence of either SspA or SspB, correspondingly, yet did not yield an evident phenotype (Additional file 10: Table S6, Additional file 12: Figure S6, and Additional file 13: Figure S7), suggesting that under the tested conditions, these residues play a less significant role in spore revival.

### Phosphorylation of key translational components influences spore revival and vegetative growth

Rapid resumption of translation is crucial for spore revival, occurring early on during the process [22]. Our quantitative analysis of the reviving spore proteome revealed a significant increase in the level of at least 100 proteins already after 10 minutes into revival (Additional file 14: Table S7), indicative of active protein synthesis. This trend continued into the 30-minute time point, with more than 170 proteins showing increased levels (Additional file 14: Table S7). Phosphorylation of ten translational factors, including ribosomal subunits and translation initiation and elongation components, were identified in our analysis. The majority of the quantified phosphorylation sites belonging to this group (4/5) showed increased phosphorylation throughout germination (Fig. 2c; Additional file 6: Table S3).

**Fig. 5:**
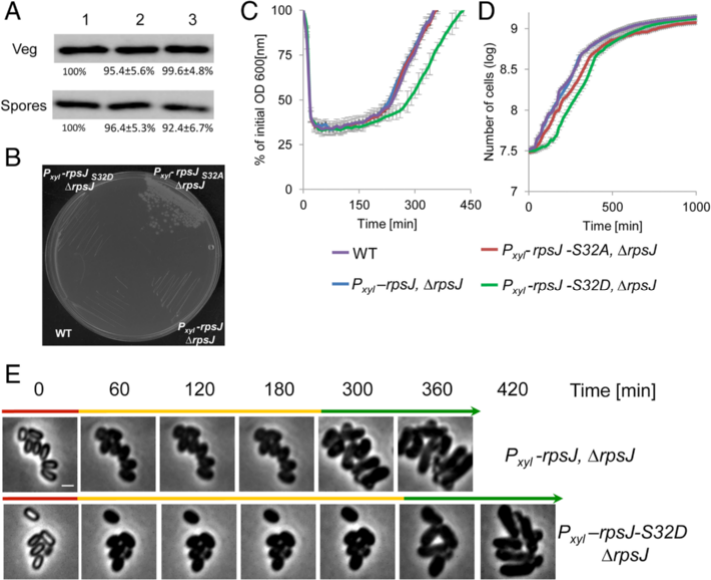
Phospho-modifications of translational factors affect spore revival and vegetative growth. **a** Proteins were extracted from either spores or vegetative cells of the strains: (1) AR223 (*Pxyl- rpsJ-HA*, ∆*rpsJ*), (2) AR224 (*Pxyl- rpsJ-S32A-HA*, ∆*rpsJ*), and (3) AR225 (*Pxyl-rpsJ-S32D-HA*, ∆*rpsJ*). Equal amounts of protein extracts were subjected to Western blot analysis. Membrane was probed with antibody against HA-tag. **b** Strains PY79 (wild type, WT), AR168 (*P*_*xyl*_ *- rpsJ*, ∆*rpsJ*), AR169 (*P*_*xyl*_ *- rpsJ-S32A*, ∆*rpsJ*), and AR185 (*P*_*xyl*_ *-rpsJ-S32D*, ∆*rpsJ*) were plated on LB supplemented with 0.5 % xylose and 7.5 μg/mL of tetracycline, incubated at 37 °C and photographed after 48 h. **c** Spores of PY79 (wild type, WT), AR168 (*P*_*xyl*_ *-rpsJ*, ∆*rpsJ*), AR169 (*P*_*xyl*_ *-rpsJ-S32A*, ∆*rpsJ*), and AR185 (*P*_*xyl*_ *-rpsJ-S32D*, ∆*rpsJ*) strains were incubated at 37 °C in S7 defined medium supplemented with L-Ala (10 mM) + 0.5 % xylose, and optical density (OD_600_) was measured at the indicated time points. The data points are averages of results obtained from three independent biological repeats. Error bars designate SD. **d** Growth curves of PY79 (wild type, WT), AR168 (*P*_*xyl*_ *-rpsJ*, ∆*rpsJ*), AR169 (*P*_*xyl*_ *-rpsJ-S32A*, ∆*rpsJ*), and AR185 (*P*_*xyl*_ *-rpsJ-S32D*, ∆*rpsJ*) strains. Cells were grown at 37 °C in S7-defined medium supplemented with L-Ala (10 mM) + 0.5 % xylose and OD_600_ was measured at the indicated time points. The data points are averages of results obtained from four independent biological repeats. Error bars designate SD. AR185 (*P*_*xyl*_ *-rpsJ-S32D*, ∆*rpsJ*) showed significantly reduced growth rates compared to the other strains by repeated measures ANOVA (*P* <0.05). **e** Spores of AR168 (*P*_*xyl*_ *-rpsJ*, ∆*rpsJ*) and AR185 (*P*_*xyl*_ *-rpsJ-S32D*, ∆*rpsJ*) strains were incubated in S7-defined medium supplemented with L-Ala (10 mM) + 0.5 % xylose and monitored by time lapse microscopy. Shown are phase-contrast images acquired at the indicated time points. Arrows signify the dominating revival stages of each population at a given time point (germination, red; ripening, orange; elongation, green). Scale bar represents 1 μm

To further establish a relationship between the phosphorylation of translational components and the progression of revival, we investigated the effect of mutating a newly identified phosphorylation site within the essential integral ribosomal protein RpsJ (small ribosomal subunit protein S10). Within the ribosome, RpsJ is located near the aminoacyl-tRNA site of the 30S subunit and interacts with 16S rRNA [44]. Our analysis revealed RpsJ to display increased phosphorylation on a previously uncharacterized site, Ser32, during spore germination (Fig. 2c; Additional file 2: Table S1 and Additional file 6: Table S3). RpsJ is highly conserved across *Bacillus* species with the Ser32 residue displaying full conservation (Additional file 15: Figure S8). Since RpsJ is an essential protein required throughout the *B. subtilis* life cycle, we were unable to introduce alleles harboring mutations at the Ser32 position at the native chromosomal locus. We therefore inserted the mutant *rpsJ-S32A/D* or the *rpsJ* wild type alleles at an ectopic site as a sole copy within the genome under the control of an inducible promoter. The constructed strains required a constant presence of the inducer to maintain viability. The mutant and native alleles presented similar expression levels and did not significantly affect spore production (Fig. 5a, Additional file 10: Table S6). Notably, it has been shown that tetracycline resistance can be conferred by mutations in RpsJ [45, 46]. Assessing the sensitivity of the phosphorylation-site mutants to tetracycline revealed the *rpsJ-S32A* phosphorylation mutant to exhibit a substantial increase in resistance to the drug, indicating a modified function, while no such effect was observed for *rpsJ-S32D* (Fig. 5b).

Analyzing spore revival kinetics utilizing OD_600_ measurements showed a clear extension of the ripening period by approximately 80 minutes for the strain harboring the *rpsJ-S32D* allele (Fig. 5c). This phenotype was further corroborated using time lapse microscopy, which uncovered an extension of the ripening period and consequently delayed outgrowth of this mutant strain (Fig. 5c, e). Yet, the *rpsJ-S32A* allele did not impact any of the revival stages (Fig. 5c). Given the essential role of RpsJ, we also assessed if and how the *rpsJ* phospho-site mutants affect vegetative growth. Both mutants displayed altered vegetative growth phenotypes with *rpsJ-S32A* showing slower growth kinetics and *rpsJ-S32D* exhibiting an extended lag period (Fig. 5d). In this regard, it is tempting to draw a comparison between the lag phase and the ripening period, as both precede cell growth. Taken together, these findings support the notion that phosphorylation of RpsJ-S32 alters rather than abolishes the activity of this protein.

We next examined the function of novel phospho-modifications detected on two additional essential translational components, namely the elongation factors EF-G (Tyr339) and elongation factor-TU (EF-TU; Tyr270), which catalyze the translocation of the tRNA and mRNA during polypeptide elongation. Amino acid substitutions at these sites did not affect spore production or spore revival kinetics (Additional file 10: Table S6 and Additional file 16: Figure S9A and S9C). However, the modified proteins EF-G-Y339A/D and EF-TU-Y270D perturbed vegetative growth (Additional file 16: Figure S9B and S9D), signifying the importance of these sites for the functionality of the proteins. Thus, the concurrent phospho-modification of multiple translational factors suggests that these alterations are important for reestablishing protein synthesis during revival.

### The phosphorylation state of HPr, the master regulator of carbon metabolism, plays a key role in spore revival

Upon revival, the spore has to instantly reinitiate metabolic activity in order to successfully resume a vegetative life form [16]. The observed dynamic phosphorylation of proteins involved in carbon metabolism (Fig. 2c; Additional file 6: Table S3), could represent a strategy to rapidly modulate these factors to produce energy for fueling the revival process. The protein exhibiting one of the greatest increases in phosphorylation upon germination is HPr, the master regulator of carbon metabolism in *B. subtilis* [47]. HPr serves a dual function; on the one hand it is part of the phosphoenolpyruvate sugar phosphotransferase system (PTS), governing carbohydrate transport metabolism and utilization [48], on the other, it functions as an allosteric effector of the transcriptional regulator carbon catabolite protein A (CcpA) [47]; the latter transcription regulatory process is termed carbon catabolite repression (CCR) [49]. We identified and quantified phosphorylation events occurring on two previously known HPr sites: Ser12, the function of which has not been revealed [29, 50], and Ser46, which modulates the PTS and the CCR activities [47]. HPr-S12 did not show a significant change in phosphorylation level at the onset of revival, whereas HPr-S46 exhibited a sharp increase in phosphorylation 10 minutes post revival induction that remained stable at the subsequent 30 minute time point (Fig. 2c; Additional file 6: Table S3). This boost in phosphorylation levels coincided with the presence of two PTS sugars, glucose and fructose, in the revival medium.

**Fig. 6:**
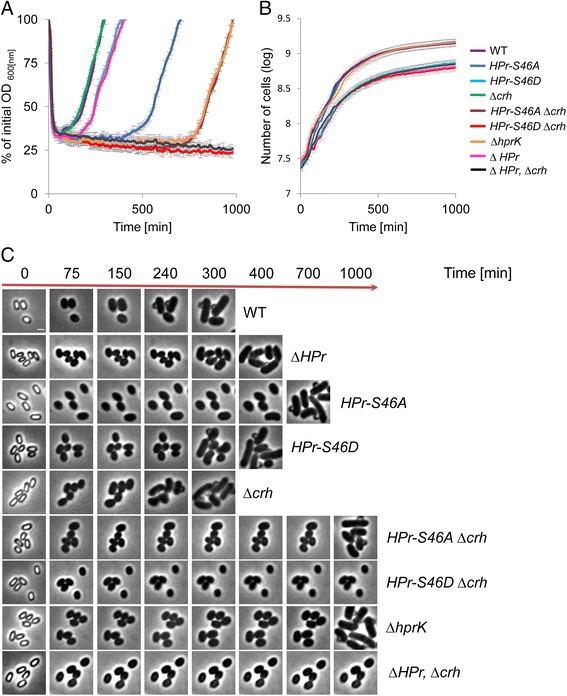
The phosphorylation state of HPr, the master regulator of carbon metabolism, is crucial for spore revival. **a** Spores of PY79 (wild type, WT), AR213 (*HPr-S46A*), AR214 (*HPr-S46D*), AR88 (∆*crh*), AR129 (*HPr-S46A*, ∆c*rh*), AR130 (*HPr-S46D*, ∆*crh*), AR127 (∆*HPr*), AR128 (∆*HPr*, ∆c*rh*), and AR196 (∆*hprK*) strains were incubated at 37 °C in S7-defined medium supplemented with L-Ala (10 mM) and glucose as a sole carbon source, and optical density (OD_600_) was measured at the indicated time points. The data points are averages of results obtained from three independent biological repeats. Error bars designate SD. **b** Growth curves of PY79 (wild type, WT), AR213 (*HPr-S46A*), AR214 (*HPr-S46D*), AR88 (∆*crh*), AR129 (*HPr-S46A*, ∆c*rh*), AR130 (*HPr-S46D*, ∆*crh*), AR127 (∆*HPr*), AR128 (∆*HPr*, ∆c*rh*), and AR196 (∆*hprK*) strains. Cells were grown at 37 °C in S7-defined medium supplemented with L-Ala (10 mM) and glucose as a sole carbon source, and OD_600_ was measured at the indicated time points. The data points are averages of results obtained from three independent biological repeats. Error bars designate SD. **c** Spores of PY79 (wild type, WT), AR213 (*HPr-S46A*), AR214 (*HPr-S46D*), AR88 (∆*crh*), AR129 (*HPr-S46A*, ∆c*rh*), AR130 (*HPr-S46D*, ∆*crh*), AR127 (∆*HPr*), AR128 (∆*HPr*, ∆c*rh*), and AR196 (∆*hprK*) strains were incubated in S7-defined medium supplemented with L-Ala (10 mM) and glucose as a sole carbon source, and monitored by time lapse microscopy. Shown are phase-contrast images acquired at the indicated time points. Arrows signify the dominating revival stages of each population at a given time point (germination, red; ripening, orange; elongation, green). Scale bar represents 1 μm

The physiological role of HPr-S46 phosphorylation has been extensively studied during vegetative growth, where it has been shown to affect sugar uptake [47]. HPr-S46 phosphorylation also facilitates the interaction of HPr with CcpA, enabling binding to regulatory sequences. Accordingly, HPr-S46A abolishes CCR while, S46D induces permanent CCR and perturbs carbohydrate uptake [51]. Given the critical regulatory role of this site during vegetative growth, we examined its impact on spore revival in a defined medium containing glucose as a sole carbon source. Noticeably, strains producing the HPr-S46A or HPr-S46D mutant proteins exhibited reduced sporulation efficiency (Additional file 10: Table S6). Moreover, the HPr-S46A and HPr-S46D mutant spores exhibited delayed revival patterns. The HPr-S46A mutant displayed a 400-minute extension of the ripening period, whereas HPr-S46D mutant displayed a moderate extension of 100 minutes (Fig. 6a, c). Surprisingly, no correlation was observed between vegetative growth and revival dynamics, as HPr-S46A mutant grew similarly to the wild type, while HPr-S46D exhibited a significant growth perturbation (Fig. 6b). Thus, the HPr phosphorylation state has different implication on the revival process versus vegetative growth.

*B. subtilis* possesses an HPr homologue called carbon-flux regulating HPr (Crh) that is part of CCR regulation but is not involved in PTS activity [52]. Therefore, we next examined the effect of the HPr-S46A/D mutant proteins in the absence of *crh*. As can be seen in Fig. 6a, c, *crh* deletion resulted in a more pronounced HPr mutant phenotype, with profound extension of the ripening period (700 minutes) for spores harboring HPr-S46A. Furthermore, no completion of revival was observed for spores harboring HPr-S46D at the course of 1000 minutes after revival induction. Exit from the ripening period was eventually detected after approximately 1400 minutes (data not shown). These observations emphasize the importance of CCR in the revival process. Consistent with these findings, the absence of the ATP-dependent kinase/phosphatase HprK, required for phosphorylation of both HPr and Crh [53], resulted in revival phenotypes similar to those displayed by cells expressing HPr-S46A and lacking Crh (Fig. 6a, c). Once again, *HPr-S46A* ∆*crh* or ∆*hprK* mutants did not display any growth phenotypes (Fig. 6b), suggesting that unique constraints are imposed during revival. There was also no correlation between sporulation efficiency and revival dynamics among the HPr mutant strains, implying that the observed phenotypes were revival-specific and not a result of perturbed spore production.

The level of phosphorylation at HPr-S46 is dependent on the utilized carbon source [54]. Therefore, we next asked whether replacing glucose with other carbon sources would alter the revival dynamics of the HPr-S46A/D mutants. Consequently, we repeated the revival experiments in the presence of the PTS sugar mannose [55]. HPr mutants, in both wild type and ∆*crh* background, displayed a prominent delay in exiting the ripening period (Additional file 17: Figure S10A). Furthermore, the HPr-S46D mutant protein elicited an additional defect in the outgrowth phase (Additional file 17: Figure S10A), which coincided with a perturbation in vegetative growth (Additional file 17: Figure S10B).

Taken together, HPr-S46 phosphorylation state has a profound effect on the ability of the spore to revive, and this phenotype is emphasized in the absence of Crh. Moreover, the aberrant revival kinetics is not limited to glucose and can be monitored when alternative PTS sugars are utilized.

## Discussion

**Fig. 7:**
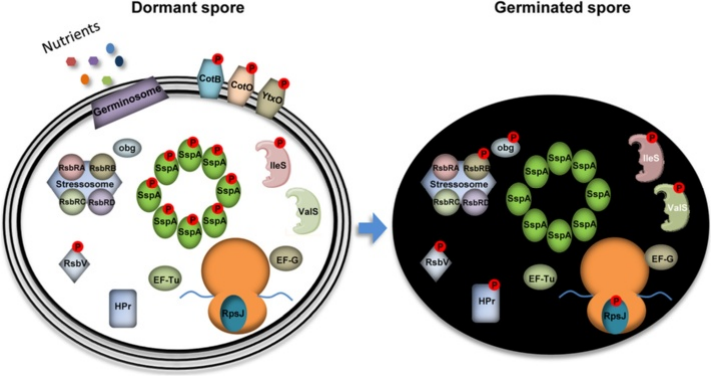
Protein phosphorylation events occurring during spore germination. A model depicting protein phosphorylation events occurring during spore germination based on the overall data obtained in our analysis. Left and right panels represent the dormant and the germinated spore phosphoproteomes, respectively. Proteins having phospho-sites lacking quantitative data were included without indicating their phosphorylation state. Proteins that appear only in the spore are degraded during germination

Spore revival is a unique developmental process enabling the study of cell conversion from a quiescent to a fully active state. The resumption of metabolic activity upon spore germination, the earliest revival step, has been traditionally ascribed to rehydration, increase in pH, and changes in ionic levels within the spore core. Herein, we defined the germinating spore phosphoproteome, and found it to be mainly composed of newly identified phosphorylation sites located within proteins involved in basic biological functions, suggestive as of their potential role in the rapid triggering of metabolic re-acquisition (Fig. 7). The magnitude of changes in phosphorylation levels between the dormant and germinating spore phosphoproteomes, reinforces the view that phospho-modifications could play a key role in rapidly modulating protein activity throughout this cellular conversion. Furthermore, we evidenced a causative relationship between phosphorylation dynamics and the progression of spore revival by demonstrating that spores bearing mutations at specific phosphorylation sites within proteins, representative of key biological processes, exhibit aberrant spore revival phenotypes. Our analysis may provide a basis for future detailed investigation of phosphorylation events occurring throughout the complete revival timeline.

Phospho-modifications of Ser/Thr sites constituted the majority of events defined in this study. Furthermore, our phosphoproteome indicated that the two known Ser/Thr kinases, PrkC and YabT, are phosphorylated during the process. PrkC was shown to be a kinase receptor mediating germination induced by muropeptides [14], while YabT has recently been shown to take part in the regulation of sporulation [56]. Deleting each kinase separately or in parallel did not yield a detectable phenotype upon revival (Additional file 18: Figure S11), suggesting that they cannot account for the phosphorylation phenotypes described above. It is tempting to speculate that *B. subtilis* possesses additional, as yet undetermined, kinases, as previous phosphoproteomic studies have shown that known bacterial kinases cannot account for all the detected phosphorylation events [57, 58]. An alternative but not mutually exclusive scenario is that the unique and dramatic physiological changes occurring during the course of germination promote multiple autophosphorylation events.

The vegetative *B. subtilis* phosphoproteome was recently shown to be highly dynamic during exponential growth, with the most significant changes occurring upon entry into the stationary phase [30]. Interestingly, the majority of phospho-site mutations investigated in this study resulted in extension of the ripening period, which serves as the transition phase between a germinating spore and a vegetatively growing cell. Thus, it is conceivable that Ser/Thr/Tyr phosphorylation is a general strategy undertaken by *B. subtilis* to modulate its activity during transition periods. However, Ser/Thr/Tyr phosphorylation events likely represent only a fraction of the germinating spore phosphoproteome. In fact, His/Asp phosphorylations, through which two component and phospho-relay systems exert their functions, have been established as major sensory elements affecting bacterial physiology. Such systems were shown to induce chemotaxis, sporulation, and competence in response to environmental stimuli [59]. His/Asp phosphorylations are highly labile and therefore not amenable to MS analysis at this stage [60]. Recent studies have revealed an additional phospho-modification located on Arg residues [31, 32]. Notably, several of the phospho-modified proteins monitored in our study have also been shown to contain phosphorylated Arg sites [31, 32]. Thus, it is plausible that co-occurring phosphorylation events taking place during germination provide the driving force for spore revival, with the phosphorylation events defined here reflecting the significance and complexity of the process.

The phosphoproteome of the reviving *B. subtilis* spore includes a large group of proteins unique to sporulation and conserved among *Bacillus* species. Within this group, we could identify phospho-sites conserved only across a narrow number of *Bacillus* species. Notably, different *Bacillus* species require dissimilar germinants to initiate revival [61, 62]. Unique phospho-modifications may therefore delineate distinctive germination pathways that reflect the diverse physiological requirements of a given species residing in a specific ecological niche. On the other hand, some of the identified phospho-sites are highly conserved in *Bacillus* species, suggesting a more general role for these sites in spore formers. The SspA-S47 (and its equivalent SspB-S45) position investigated in this study is among the most conserved sites, showing dephosphorylation trends at the course of germination. Mutating this position resulted in profound phenotypes, such as high UV sensitivity of dormant and germinating spores and extension of the ripening period accompanied with prolonged persistence over the chromosome, all consistent with higher affinity to DNA [42, 43]. In this way, phosphorylation provides the spore with an additional layer of regulation over the Ssp-DNA interaction during sporulation and spore revival. An alternative, but not mutually exclusive, regulatory mechanism could be mediated by other phosphorylation sites at the N- and C-termini of both SspA and SspB. Ssp-DNA and Ssp-Ssp interactions were shown to be dependent on the overall charge of these domains, which is likely to be affected by phosphorylation [43, 63].

During the transition into a growing cell, the spore has to rapidly reactivate core vegetative systems. Consistently, we have recently shown that translation initiates already during germination [15]. Our phosphoproteomic data, in conjunction with primary functional analysis, support the view that the awakening of the translational machinery is mediated, at least in part, by an array of phospho-modification events activating central translational factors. Regulation of translation has long been shown to be mediated by phospho-modifications of various translational components in both eukaryotic and prokaryotic organisms [64, 65]. Furthermore, EF-G was found to undergo phosphorylation during germination triggered by muropeptides [14]. Herein, we identify EF-G as well as nine additional translational components, as harboring phospho-sites. Overall, our data imply that translation activation during spore revival relies on multiple parallel modifications, which evidently also serve a function in subsequent vegetative growth.

Carbon utilization is among the systems that need to be activated most immediately upon revival. We established a central role in spore revival for the phosphorylation state of the master regulator of carbon metabolism, HPr. HPr-S46 phosphorylation affects cell metabolism at multiple levels exerting its effect through CCR, inducer exclusion, as well as phosphorylation of transcription regulators and several metabolic enzymes [47]. We provide evidence that the well characterized HPr-S46 phospho-modification is crucial to facilitate revival. Since the effect of mutating the S46 site is exacerbated in the absence of crh, it is feasible that CCR is key for exiting dormancy. In fact, many genes that are regulated through HPr appear to be differentially regulated during spore revival [66]. Future comprehensive parallel analyses entailing transcriptomics, proteomics, and metabolomics will be necessary to reveal the exact molecular events occurring throughout the process.

Some of the phenotypes associated with mutations in phosphorylation sites investigated in this study could be attributed to general changes in protein properties. Nevertheless, some of the observed phenotypes were exclusive to either exit from dormancy or vegetative growth, suggesting that the constructed mutations indeed recapitulated the phosphorylation state of the examined proteins. Furthermore, this observation reinforces the view that spore revival is a developmental process regulated by unique molecular factors alongside constituents common to other stages in the *B. subtilis* life cycle. The findings that the phospho-site mutants designed and investigated here mainly affect the extent of the ripening period, suggest that this phase has unique spore-specific features. The identification of phosphorylation dynamics as critical for this developmental transition could serve as a platform to design phosphorylation inhibitors to combat spore forming pathogens.

## Conclusions

In this research, we utilized *B. subtilis* as a model organism to comprehensively study the Ser/Thr/Tyr phosphoproteome of a germinating bacterial spore. We present first evidence that widespread dynamic changes occur upon induction of germination. Furthermore, by mutating select phosphorylation sites within proteins playing part in key biological processes (translation, carbon metabolism, and spore-specific determinants), a functional link between phosphorylation and the progression of spore revival was revealed.

## Methods

### Strains and general methods

*B. subtilis* strains are listed in Additional file 19: Table S8, and their construction is described in Additional Methods. Plasmids and primers used for this study are described in Additional file 19: Table S9 and Additional file 19: Table S10, respectively.

### Spore preparation and purification

Sporulation of various strains was induced by exhaustion in liquid CDSM medium [67] (40 mM 3-(N-Morpholino)-propanesulfonic acid (MOPS) pH 7, 4 mM KH_2_PO_4_, 9.5 mM (NH_4_)_2_SO_4_, 5 mM L-lactic acid, 8 mM L-glutamic acid, 1× MT mix, 20 mM D-glucose). Spore formation was assessed by heat resistant colony forming units. Cells were induced to form mature spores by incubating them in CDSM medium at 37 °C for 36 h. The spore culture was diluted and 100 μL of the desired dilutions were plated in duplicates on LB plates. The remaining dilutions were further incubated for 30 min at 65 °C, and the same diluted samples were plated again in duplicates on LB plates. All plates were incubated for 24 h at 37 °C and colony forming units (CFU) were determined. Sporulation efficiency was evaluated by comparing the number of CFU before and after heat treatment. Spores were purified using a three-step Histodenz (Sigma) gradient. Briefly, a 10-mL 36 h CDSM culture was washed in distilled water and resuspended in 1 mL of 20 % Histodenz solution for 30 min on ice. Spores were then placed on top of a two-step gradient made up from 2 mL 40 % Histodenz on top of 6 mL 50 % Histodenz. After centrifugation (90 min, 4,000 rpm, 23 °C, Eppendorf A-4-62 rotor), the pellet containing >99 % free spores as evaluated by phase-contrast microscopy was washed three times with distilled water to remove residual Histodenz.

### Quantitative phosphoproteomic analysis

For phosphoproteomic analysis, purified spores were diluted to an OD_600_ of 0.5 in reviving medium containing MOPS (40 mM, pH 7), AGFK (2.5 mM L-aspargine, 5 mg/mL D-glucose, 5 mg/mL D-fructose, 50 mM KCl) and L-ala (10 mM) [68], and germinated for 30 min. Samples from t = 0, 10, and 30 min were collected by centrifugation, resuspended in 50 mM Tris–HCl (pH 7.4) supplemented with 0.05 % SDS, Halt Protease and phosphatase inhibitors (Pierce), and lysed using Fastprep (FastPrep (MP) 6.5, 60 sec, ×3). Following cell lysis, the supernatant was additionally centrifuged at 100,000 *g* for 30 min to remove cell wall remnants and other insoluble matter. The protein suspension was precipitated using acetone. The resulting protein extracts were resuspended in denaturation buffer (6 M urea/2 M thiourea in 10 mMTris). The amounts of protein present from all three time points were determined through utilizing the Bradford assay (Bio-Rad, Munich, Germany). Approximately 6 mg of protein extract from each time point were digested into peptides as previously described [29] with slight modifications: proteins were reduced with 1 mM dithiothreitol, followed by an alkylation step with 5.5 mM iodacetamide. The peptide mixture was pre-digested with endoproteinase Lys-C (Waco, Neuss, Germany) for 3 h at room temperature (RT) followed by an overnight digestion step with trypsin at RT. For proteome analysis, approximately 10 μg of peptides from each sample were acidified to pH 2.5 and desalted using C_18_ stage tips [69], followed by nano-LC-MS/MS analysis. The remaining peptides from each sample were subsequently enriched for phosphopeptides using TiO_2_ chromatography as previously described [70] with additional modifications. TiO_2_ beads with a diameter of 10 μm (MZ Analysetechnik, Mainz, Germany) were incubated with 2,5-dihydrobenzoic acid in 80 % acetonitrile at a concentration of 30 mg/mL. Approximately 5 mg of TiO_2_ beads were added to each sample and incubated for 20 min, while being slightly agitated (at RT). This procedure was repeated consecutively for a total of five times. For each round of TiO_2_ enrichment, beads underwent a washing step with 1 mL of 30 % acetonitrile/ 3 % trifluoroacetic acid and 80 % acetonitrile/0.1 % trifluoroacetic acid for a period of 10 min. Enriched phosphopeptides were eluted from the TiO_2_ beads using 100 μL of 40 % ammonium hydroxide solution in 60 % acetonitrile, pH 10.5. The five elutions from each phosphopeptide enrichment step were then pooled together in order to yield one sample per experimental condition (t = 0, 10, and 30 min). The sample volume was reduced in a vacuum centrifuge at RT to 6 μL followed by nano-LC-MS/MS analysis. Three phosphoproteome measurements were performed; one was purely qualitative, one involved 15 N labeling of bacterial culture [71], and one utilized label-free quantification. For technical reasons, 15 N-labeling was not used for quantification, but only for identification of phosphorylation events. Of note, SILAC experiments were not applicable since strains were auxotrophs for Lys or Arg sporulate poorly, yielding very low amounts of mature spores.

Nano-LC-MS/MS was performed on an EasyLCnano-HPLC (Proxeon Biosystems, Odense, Denmark) coupled to the LTQ Orbitrap XL mass spectrometer. Peptides were loaded onto a 15-cm nano-HPLC column (inner diameter: 75 μm, tip diameter: 8 μm) in-house packed with reverse-phase 3 μm C18 spheres (Dr. Maisch, Ammerbuch, Germany). Peptides were loaded onto the column with 0.5 % acetic acid at a flow rate of 500 nL/min. A linear, segmented 130 min gradient consisting of 5–33 % of Solvent B (80 % acetonitrile in 0.5 % acetic acid) was applied at a constant flow rate of 200 nL/min. Separated peptides were ionized through electrospray ionization using the electrospray ion source (Proxeon Biosystems). The OrbitrapXL was operated in the data-dependent positive ion mode utilizing the following acquisition cycle: one survey scan in the Orbitrapmass analyzer was acquired at the range of m/z 300–2,000 Thompson (Th) at a resolution of 60,000 (defined at m/z = 400) with a target value of 1 × 10^6^ charges, followed by fragmentation via collision induced dissociation of the 10 most intense precursor ions in the linear ion trap analyzer (LTQ) at a target value of 5× 10^3^ charges. Dynamic exclusion of precursor ions was applied for 90 sec. Additionally, the lock-mass option was enabled for internal calibration [72]. For MS analysis of enriched phosphopeptides, the LTQ Orbitrap XL was utilized with additional parameters: collision induced dissociation was performed on the five most intense precursor ions. Moreover, the multi stage activation feature was enabled in all MS/MS events where a neutral loss was detected at masses of singly (−97.97 Th), doubly (−48.99 Th), and triply (−32.66 Th) charged precursor ions. For each experimental condition, both proteome and phosphoproteome samples were analyzed in triplicate measurements in order to be compatible with LFQ analysis.

In terms of both processing and analyzing the MS data, all acquired MS RAW files were processed together using the MaxQuant software suite version 1.2.2.9 [27]. Database search was performed using the Andromeda platform, which is built into Maxquant [28] against a target-decoy database of *B. subtilis* 168 obtained from Uniprot (taxonomy ID 1423), containing a total of 4,195 *B. subtilis* protein entries and 262 contaminant proteins. Briefly, trypsin was fixed as the protease with a maximum allowance of two missed cleavages. Variable modifications were set for methionine oxidation, N-terminal acetylation, and phosphorylation on serine, threonine, and tyrosine residues. Cysteine carbamidomethylation was set as a fixed modification. A mass tolerance was set to 6 ppm and 0.5 Daltons for MS and MS/MS scans, respectively. A false discovery rate of 1 % was applied at the peptide, protein, and phosphorylated site level. Finally, the LFQ algorithm was set to infer quantitative information. Phosphorylation sites with a localization probability of ≥0.75 were considered to be localized. MS/MS spectra of phosphorylated peptides were filtered through manual validation in order to ensure high data quality. Phosphorylation site intensities were normalized with their respective protein intensities in order to determine if a bias was present based on the changing protein abundance. After this normalization step, phosphorylation sites were considered as significantly changing, based on the intensity-weighed statistical test known as “Significance B” (*P* = 0.05). Finally, protein GO annotation and clustering analysis was done using DAVID and Subtiwiki [73, 74]. The mass spectrometry data was deposited to the ProteomeXchange Consortium via the PRIDE partner repository dataset (PXD002559).

### Spore revival measurements

For spore revival experiments, purified spores were heat activated (65 °C, 30 min), diluted to an OD_600_ of 0.5 in S7 medium (100 mM MOPS pH 7, 5 mM potassium phosphate buffer pH 7, 10 mM (NH_4_)_2_SO_4_, 20 mM L-glutamic acid, 1× MT mix, 2 % D-glucose) supplemented with 10 mM L-Ala [75], and divided into a 96-well plate at a final volume of 0.2 mL of culture per well. An OD_600_ was followed by Wallac Victor 2 multiwell fluorometer (Perkin Elmer) at 37 °C set with constant shaking (3 mm orbital, fast speed).

### Vegetative growth measurements

For growth curve analysis, overnight cultures were diluted to OD_600_ of 0.05 in S7 medium supplemented with L-Ala (10 mM) divided into a 96-well plate at a final volume of 0.2 mL culture per well, and an OD_600_ was followed in a Wallac Victor 2 multiwell fluorimeter at 37 °C set with constant shaking (3 mm orbital, fast speed).

### Light microscopy

Light microscopy was carried out as described previously [17]. Briefly, bacterial cells (0.2 mL) were centrifuged and resuspended in 50 μL of PBS ×1. Specimens were placed on 1 % agarose pads and visualized using an Axioplan 2 microscope (Zeiss) equipped with a CoolSnap HQ camera (Photometrics, Roper Scientific). For time-lapse revival experiments, spores were placed on 1 % agarose pads made of the S7 medium supplemented with L-ala (10 mM), and incubated in a temperature-controlled chamber (Pecon-Zeiss) at 37 °C. For GFP measurements, the intensity of a wild-type (PY79) strain, lacking the *gfp* gene, was subtracted from the net average fluorescence intensity. Samples were photographed using AxioObserver Z1 (Zeiss), equipped with CoolSnap HQII camera (Photometrics, Roper Scientific). System control and image processing were performed using MetaMorph 7.7 software (Molecular Devices).

### UV resistance assay

Purified spores were diluted to an OD_600_ of 0.5 and different samples were exposed to increasing doses of UV (254 nm) irradiation using UVC 500 Ultraviolet Crosslinker (Amersham). The irradiated spore suspensions were serially diluted and plated on LB. After an overnight incubation at 37 °C, CFU/mL culture was determined. The survival rate was calculated as percentage of the CFU/mL obtained without UV exposure.

#### UV resistance during germination assay

Purified spores (OD_600_ of 0.5) were incubated for 15 min at 65 °C. Then, spores were germinated at 37 °C in medium containing L-ala (10 mM) and MOPS (40 mM, pH 7). At various times, samples were, irradiated with UV (254 nm) using UVC 500 Ultraviolet Crosslinker (at energy setting 500 mj/cm^2^; Amersham). The irradiated spores were serially diluted and plated on LB. After an overnight incubation at 37 °C CFU/mL culture was determined. The survival rate was calculated percentage of the CFU/mL obtained at t = 0 minutes prior to UV exposure.

### Western blot analysis

Proteins were extracted from dormant spores and vegetative cells as described for phosphoproteomic analysis experiments. Extracts were incubated at 100 °C for 10 min with Laemmli sample buffer. Proteins were separated by SDS-PAGE 12.5 % and electroblotted onto a polyvinylidene difluoride transfer membrane (Immobilon-P; Millipore). For Immunoblot analysis of HA tag fusion proteins, membranes were blocked for 1 h at room temperature (0.05 % Tween-20, 5 % skim milk in TBS ×1). Blots were then incubated for 1 h at room temperature with polyclonal rabbit anti-HA antibodies (Sigma; 1:10,000 in 0.05 % Tween-20, 5 % skim milk in TBS ×1). Next, membranes were incubated for 1 h at RT with peroxidase conjugated goat anti-rabbit secondary antibody (Bio-Rad; 1:10,000 in 0.05 % Tween-20, 5 % skim milk). EZ-ECL kit (Biological Industries, Beit Haemek, Israel) was used for final detection. Band intensities were quantified by comparing the total intensity of identical-sized regions using MetaMorph 7.7 software (Molecular Devices) on the immunoblot image.

Additional Methods including strain construction, are described in (Additional file 19: Supporting Methods).

### Availability of supporting data

MS/MS raw data files have been uploaded to the ProteomeXchange Consortium via the PRIDE partner repository (http://www.proteomexchange.org/databases/pride) and are freely available for download and analysis under dataset PXD002559.

## Additional files

Additional file 1: Figure S1. Reproducibility of measured protein ratios within three technical replicates. (A) Phosphoproteome measurements. (B) Proteome measurements. Pearson correlation coefficient was higher than 0.95 in all cases. (PDF 503 kb) Additional file 2: Table S1. The germinating spore phosphoproteome. Phospho-sites identified in this study were clustered according to their biological processes, as assigned by the DAVID functional annotation tool. (XLSX 27 kb) Additional file 3: Figure S2. Comparison of germinating spore and vegetative growth phosphoproteomes. (A) Overlap between the germinating and vegetative phosphoproteins. (B) Common phosphorylation sites between the germinating and vegetative phase phosphoproteins. (C) The identity of the overlapping 17 phosphorylation sites and the stage of vegetative growth at which they show increased phosphorylation. (PDF 185 kb) Additional file 4: Table S2. Phosphorylation sites detected in Ravikumar et al. [30] and current study. Comparison between phosphorylation sites detected during *B. subtilis* vegetative growth and germination. (XLSX 13 kb) Additional file 5: Figure S3. Overlap in identification of phosphorylated proteins/phospho-sites and distribution of measured protein ratios with assessment of statistical significance. (A) Venn diagram representing the overlap in identification of phosphorylated proteins between the three biological experiments. (B) Venn diagram representing the overlap in identification of phospho-sites between the three biological experiments. (C) Changes in 10 min germinating spore proteome. (D) Changes in 30 min germinating spore proteome. Significantly changing ratios are depicted in red (Significance B, *P* ≤0.05). (PDF 414 kb) Additional file 6: Table S3. Dynamics of the germinating spore phosphoproteome. Changes in protein phosphorylation level between 0 to 10- and 30-minute time points. (XLSX 16 kb) Additional file 7: Table S4. Estimated occupancies of phosphorylation sites detected in this study. (XLSX 44 kb) Additional file 8: Table S5. Deletion- and site-directed mutants assessed in this study. (PDF 206 kb) Additional file 9: Figure S4. SspA structure and conservation across *Bacillus* species. (A) Multiple sequence alignment of *B. subtilis* SspA and its homologue proteins from representative *Bacillus* species. Conserved Ser47 residue is highlighted in solid red and other Ser residues are boxed in purple. The corresponding conservation level and consensus sequence are shown below. The multiple sequence alignment was constructed using Jalview. (B) Ribbon diagram of SspA (aa 12–65) protein (cyan) with bound DNA (pink). Ser47 (red) is located at the tip of the second alpha helix. The N and C protein terminals are absent from the structure. Protein structure was predicted by SWISS-MODEL (http://swissmodel.expasy.org/). (PDF 205 kb) Additional file 10: Table S6. Sporulation efficiency of strains used in this study. (PDF 102 kb) Additional file 11: Figure S5. Characterization of SspB-S45 phosphorylation mutants. (A) Spores of PY79 (WT), AR227 (*sspB-S45A*), AR228 (*sspB-S45D*), AR229 (*sspA-S47A*, *sspB-S45A*), AR230 (*sspA-S47D*, *sspB-S45D*), AR231 (*sspB-S45A*, ∆*sspA*), AR232 (*sspB-S45D*, ∆*sspA*), AR179 (∆*sspA*), AR186 (∆*sspB*), and AR195 (∆*sspA*, ∆*sspB*) strains were incubated in S7- supplemented with L-Ala (10 mM) at 37 °C, and optical density (OD_600_) was measured at the indicated time points. Data are presented as a fraction of the initial OD_600_ of the phase-bright spores. (B) Spores of PY79 (WT), AR227 (*sspB-S45A*), AR228 (*sspB-S45D*), AR229 (*sspA-S47A*, *sspB-S45A*), AR230 (*sspA-S47D*, *sspB-S45D*), AR231 (*sspB-S45A*, ∆*sspA*), AR232 (*sspB-S45D*, ∆*sspA*), AR179 (∆*sspA*), AR186 (∆*sspB*), and AR195 (∆*sspA*, ∆*sspB*) strains were exposed to increasing UV (254 nm) doses (mj/cm^2^) and plated on LB for viable count. Percentage survival was calculated by dividing the viable spore titer at any UV dose (mj/cm^2^) with the spore titer obtained from the non-irradiated spores. (C) Spores of PY79 (WT), AR227 (*sspB-S45A*), AR228 (*sspB-S45D*), AR229 (*sspA-S47A*, *sspB-S45A*), AR230 (*sspA-S47D*, *sspB-S45D*), AR231 (*sspB-S45A*, ∆*sspA*), AR232 (*sspB-S45D*, ∆*sspA*), AR179 (∆*sspA*), AR186 (∆*sspB*), and AR195 (∆*sspA*, ∆*sspB*) strains were germinated with L-Ala (10 mM). Samples were taken at the indicated time points, irradiated with 500 mj/cm^2^ UV (254 nm) and plated on LB. Percentage survival was calculated by dividing the viable spore titer at any given time point with the spore titer obtained from spores irradiated at the 0 time point. The data points are averages of results obtained from three independent biological repeats. Error bars designate SD. For simplicity, strains lacking *sspA* were not depicted in B and C due to their high UV sensitivity (Fig. 3). (PDF 281 kb) Additional file 12: Figure S6. Characterization of SspA-S6,S9,S58 phosphorylation mutants. (A) Spores of PY79 (wild type, WT), AR211 (*sspA-S6A*,*S9A*,*S58A*), and AR212 (*sspA-S6D*,*S9D*,*S58D*) strains were incubated in S7 defined medium supplemented with L-Ala (10 mM) at 37 °C, and optical density (OD_600_) was measured at the indicated time points. Data are presented as a fraction of the initial OD_600_ of the phase-bright spores. Decreasing OD_600_ signifies spore germination while increasing OD_600_ indicates spore outgrowth. The data points are averages of results obtained from three independent biological repeats. Error bars designate SD. (B) Spores of PY79 (wild type, WT), AR211 (*sspA-S6A*,*S9A*,*S58A*), and AR212 (*sspA-S6D*,*S9D*,*S58D*) strains were exposed to increasing UV (254 nm) doses (mj/cm^2^) and plated on LB for viable count. Percentage survival was calculated by dividing the viable spore titer at any given UV dose (mj/cm^2^) with the spore titer obtained from the non-irradiated spores. The data points are averages of results obtained from three independent biological repeats. Error bars designate SD. (C) Spores of PY79 (wild type, WT), AR211 (*sspA-S6A*,*S9A*,*S58A*), and AR212 (*sspA-S6D*,*S9D*,*S58D*) strains were germinated with L-Ala (10 mM). Samples were taken at the indicated time points, irradiated with 500 mj/cm^2^ UV (254 nm) and plated on LB. Percentage survival was calculated by dividing the viable spore titer at any given time point with the spore titer obtained from spores irradiated at the 0 time point. The data points are averages of results obtained from three independent biological repeats. Error bars designate SD. (D) Spores of AR186 (∆*sspB*), AR191 (*sspA-S6A*,*S9A*,*S58A*, ∆*sspB*), and AR192 (*sspA-S6D*,*S9D*,*S58D* ∆*sspB*) revival was followed as described in (A). (E) UV resistance of spores of AR186 (∆*sspB*), AR191 (*sspA-S6A*,*S9A*,*S58A*, ∆*sspB*), and AR192 (*sspA-S6D*,*S9D*,*S58D*, ∆*sspB*) was determined as described in (B). (F) UV resistance of AR186 (∆*sspB*), AR191 (*sspA-S6A*,*S9A*,*S58A*, ∆*sspB*), and AR192 (*sspA S6D*,*S9D*,*S58D*, ∆*sspB*) germinating spores was determined as described in (C). (PDF 112 kb) Additional file 13: Figure S7. Characterization of SspB-S6,S7 phosphorylation mutants. (A) Spores of PY79 (wild type, WT), AR233 (*sspB-S6A*,*S7A*), AR234 (*sspB-S6D*,*S7D*), AR235 (*sspB-S6A*,*S7A*, ∆*sspA*), AR236 (*sspB-S6D*,*S7D*, ∆*sspA*), AR179 (∆*sspA*), and AR186 (∆*sspB*) strains were incubated in S7-defined medium supplemented with L-Ala (10 mM) at 37 °C, and optical density (OD_600_) was measured at the indicated time points. Data are presented as a fraction of the initial OD_600_ of the phase-bright spores. Decreasing OD_600_ signifies spore germination while increasing OD_600_ indicates spore outgrowth. The data points are averages of results obtained from three independent biological repeats. Error bars designate SD. (B) Spores of PY79 (wild type, WT), AR233 (*sspB-S6A*,*S7A*), AR234 (*sspB-S6D*,*S7D*), AR235 (*sspB-S6A*,*S7A*, ∆*sspA*), AR236 (*sspB-S6D*,*S7D*, ∆*sspA*), AR179 (∆*sspA*), and AR186 (∆*sspB*) strains were exposed to increasing UV (254 nm) doses (mj/cm^2^) and plated on LB for viable count. Percentage survival was calculated by dividing the viable spore titer at any given UV dose (mj/cm^2^) with the spore titer obtained from the non-irradiated spores. The data points are averages of results obtained from three independent biological repeats. Error bars designate SD. (C) Spores of PY79 (wild type, WT), AR233 (*sspB-S6A*,*S7A*), AR234 (*sspB-S6D*,*S7D*), AR235 (*sspB-S6A*,*S7A*, ∆*sspA*), AR236 (*sspB-S6D*,*S7D*, ∆*sspA*), AR179 (∆*sspA*), and AR186 (∆*sspB*) strains were germinated with L-Ala (10 mM). Samples were taken at the indicated time points, irradiated with 500 mj/cm^2^ UV (254 nm), and plated on LB. Percentage survival was calculated by dividing the viable spore titer at any given time point with the spore titer obtained from spores irradiated at the 0 time point. The data points are averages of results obtained from three independent biological repeats. Error bars designate SD. For simplicity, strains lacking *sspA* were not depicted in B and C due to their high UV sensitivity (Fig. 3). (PDF 107 kb) Additional file 14: Table S7. Dynamics of the germinating spore proteome. Changes in protein level between 0 to 10- and 30-minute time points. (XLSX 147 kb) Additional file 15: Figure S8. RpsJ structure and conservation across *Bacillus* species. (A) Multiple sequence alignment of *B. subtilis* RpsJ with its homologues from representative *Bacillus* species. Conserved Ser32 residue is highlighted in solid red, the corresponding conservation level and consensus sequence are shown below. The multiple sequence alignment was constructed using Jalview. (B) Ribbon diagram of RpsJ protein Ser32 (red) is located at the tip of globular surface domain. Protein structure was predicted by SWISS-MODEL (http://swissmodel.expasy.org/). (PDF 282 kb) Additional file 16: Figure S9. Phospho-modifications of translation elongation factors affect vegetative growth. (A) Spores of PY79 (WT), AR165 (*P*_*hyper-spank*_ *-EF-G*, ∆*EF-G*), AR166 (*P*_*hyper-spank*_ *-EF-G-Y339A*, ∆*EF-G*), and AR167 (*P*_*hyper-spank*_ *-EF-G-Y339D*, ∆*EF-G*) strains were incubated at 37 °C in S7-defined medium supplemented with L-Ala (10 mM) and 0.5 mM IPTG, and optical density (OD_600_) was measured at the indicated time points. Data are presented as a fraction of the initial OD_600_ of the phase-bright spores. Decreasing OD_600_ signifies spore germination while increasing OD_600_ indicates spore outgrowth. (B) Strains listed in (A) were grown at 37 °C in S7- supplemented with L-Ala (10 mM) and 0.5 mM IPTG, and OD_600_ was measured at the indicated time points. AR166 (*P*_*hyper-spank*_ *-EF-G-Y339A*, ∆*EF-G*) and AR167 (*P*_*hyper-spank*_ *-EF-G-Y339D*, ∆*EF-G*) strains showed significantly reduced growth rates compared to the control strains by repeated measures ANOVA (*P* <0.05). C) Spores of PY79 (WT), AR157 (*P*_*xyl*_ *-EF-TU*, ∆*EF-TU*), AR158 (*P*_*xyl*_ *-EF-TU-Y270A*, ∆*EF-TU*), and AR159 (*P*_*xyl*_ *-EF-TU-Y270D*, ∆*EF-TU*) strains were incubated at 37 °C in S7- supplemented with L-Ala (10 mM) and 0.5 % xylose, and OD_600_ was measured at the indicated time points. Data are presented as a fraction of the initial OD_600_ of the phase-bright spores. (D) Strains listed in (C) were grown at 37 °C in S7- supplemented with L-Ala (10 mM) and 0.5 % xylose, and OD_600_ was measured at the indicated time points. The data points are averages of results obtained from three independent biological repeats. Error bars designate SD. AR159 (*P*_*xyl*_ *-EF-TU-Y270D*, ∆*EF-TU*) showed significantly reduced growth rates compared to the other strains by repeated measures ANOVA (*P* <0.05). (PDF 237 kb) Additional file 17: Figure S10. Analysis of HPr phospho-mutants during spore revival and vegetative growth. (A) PY79 (wild type, WT), AR213 (*HPr-S46A*), AR214 (*HPr-S46D*), AR88 (∆*crh*), AR129 (*HPr-S46A*, ∆c*rh*), AR130 (*HPr-S46D*, ∆*crh*), AR127 (∆*HPr*), AR128 (∆*HPr*, ∆c*rh*), and AR196 (∆*hprK*) strains were incubated at 37 °C in S7-defined medium supplemented with L-Ala (10 mM) and mannose as a sole carbon source, and optical density (OD_600_) was measured at the indicated time points. Data are presented as a fraction of the initial OD_600_ of the phase-bright spores. Decreasing OD_600_ signifies spore germination while increasing OD_600_ indicates spore outgrowth. The data points are averages of results obtained from three independent biological repeats. Error bars designate SD. (B) Growth curves of PY79 (wild type, WT), AR213 (*HPr-S46A*), AR214 (*HPr-S46D*), AR88 (∆*crh*), AR129 (*HPr-S46A*, ∆*crh*), AR130 (*HPr-S46D*, ∆*crh*), AR127 (∆*HPr*), AR128 (∆*HPr, ∆*c*rh*), and AR196 (∆*hprK*) strains. Cells were grown at 37 °C in S7 medium supplemented with L-Ala (10 mM) and mannose as a sole carbon source, and OD_600_ was measured at the indicated time points. The data points are averages of results obtained from three independent biological repeats. Error bars designate SD. (PDF 233 kb) Additional file 18: Figure S11. The absence of identified kinases does not affect spore revival. Spores of PY79 (wild type, WT), AR73 (∆*prkC*), AR102 (∆*yabT*), and AR114 (∆*prkC*, ∆*yabT*), strains were incubated at 37 °C in S7-defined medium supplemented with L-Ala (10 mM), and optical density (OD_600_) was measured at the indicated time points. Data are presented as a fraction of the initial OD_600_ of the phase-bright spores. Decreasing OD_600_ signifies spore germination while increasing OD_600_ indicates spore outgrowth. The data points are averages of results obtained from four independent biological repeats. Error bars designate SD. (PDF 100 kb) Additional file 19. Supporting Information. Supporting Methods, Supporting Tables S8–S10. (PDF 366 kb)

## Abbreviations

AGFK: 2.5 mM L-asparagine, 5 mg/mL D-glucose, 5 mg/mL D-fructose, 50 mM KCl
CCR: Carbon catabolite repression
CFU: Colony forming units
Crh: Carbon-flux regulating HPr
DPA: Dipicolinic acid
EF-G: Elongation factor G
EF-TU: Elongation factor TU
HPr: Histidine-containing phosphocarrier protein
LFQ: Label free quantitation
MOPS: 3-(N-Morpholino)-propanesulfonic acid
MS: Mass spectrometry
OD: Optical density
PTS: Phosphoenolpyruvate sugar phosphotransferase system
RpsJ: Ribosomal protein S10
RT: Room temperature
Ssp: Small acid-soluble spore protein
Th: Thompson
