# Supplementary figures and images for: Phosphoproteome dynamics mediate revival of bacterial spores
Source: BMC Biol. 2015 Sep 17;13:76. doi: 10.1186/s12915-015-0184-7 (PMC4574613; doi:10.1186/s12915-015-0184-7)

Figure S1A

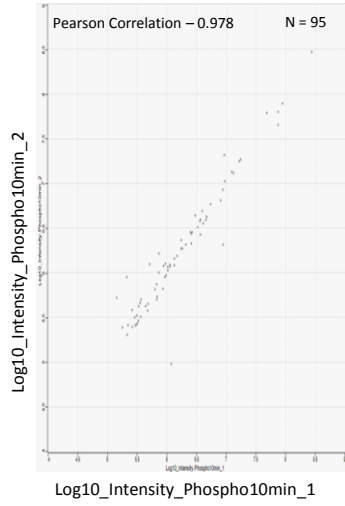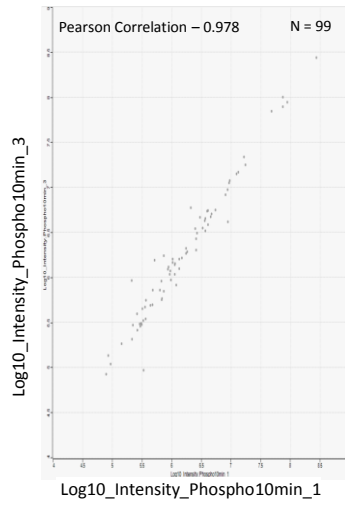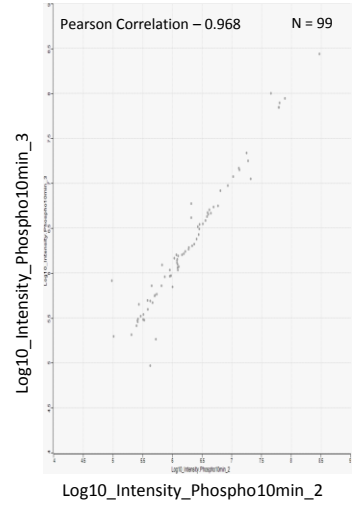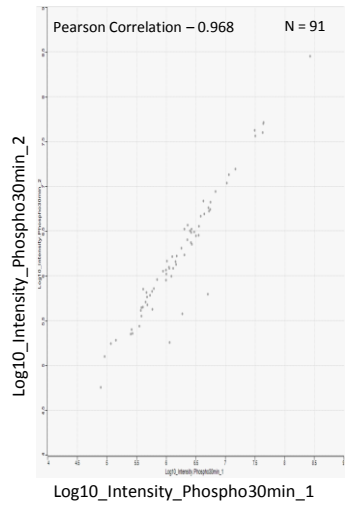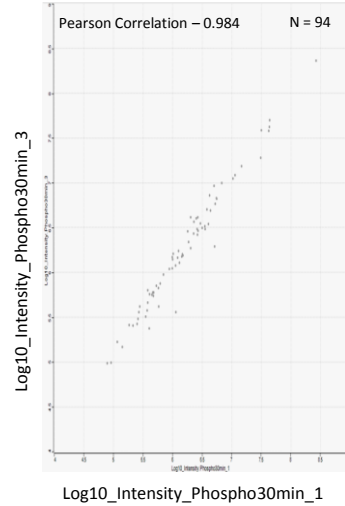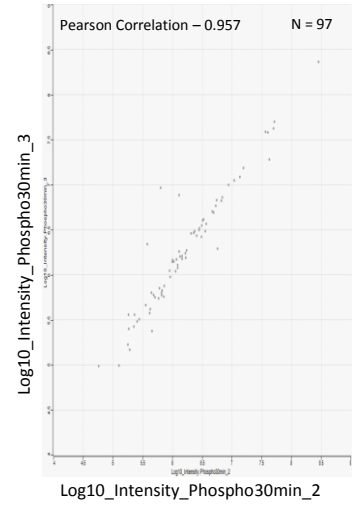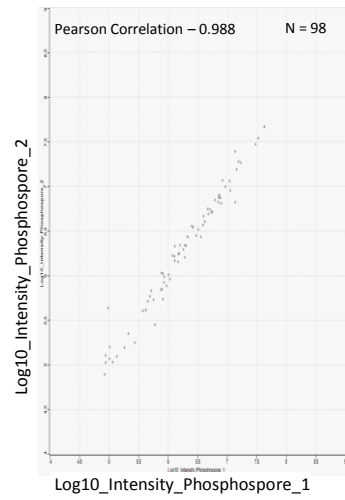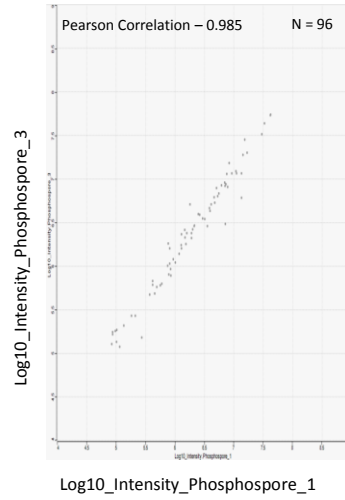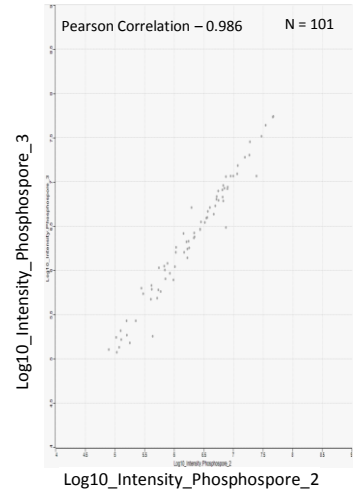

Figure S1B

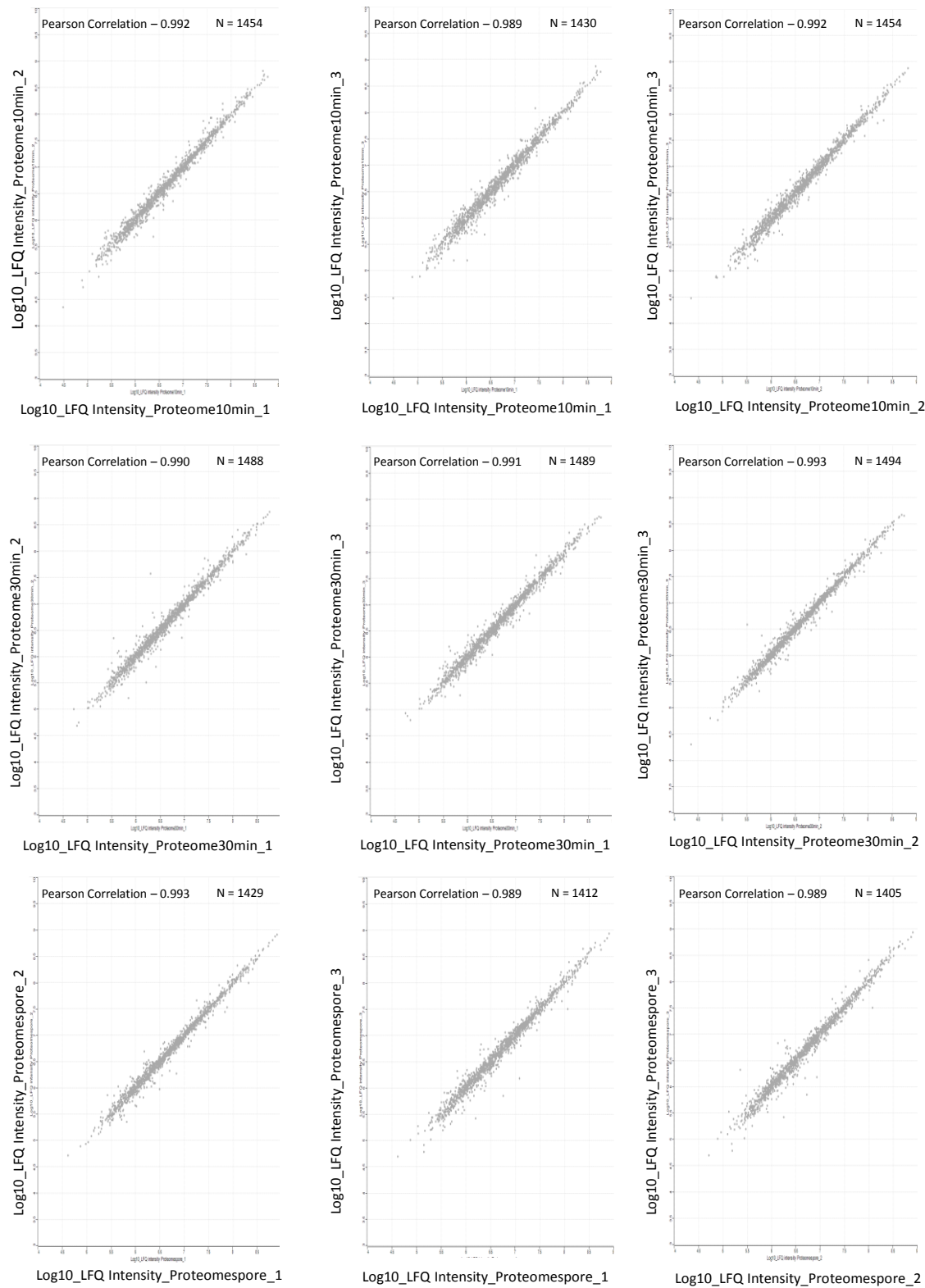

Supplement: Additional file 1: Figure S1. — Reproducibility of measured protein ratios within three technical replicates. (A) Phosphoproteome measurements. (B) Proteome measurements. Pearson correlation coefficient was higher than 0.95 in all cases. (PDF 503 kb) [file 12915_2015_184_MOESM1_ESM.pdf]

Figure S3

A. Detected Proteins

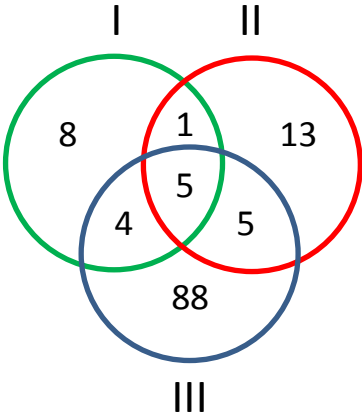

B. Detected phosphosites  
(Loc.prob.  $\geq 0.75$ )

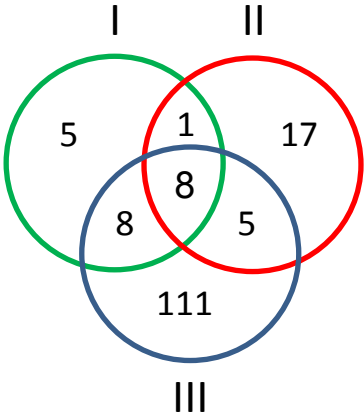

C. 10min (sigB:  $p=0.05$ )

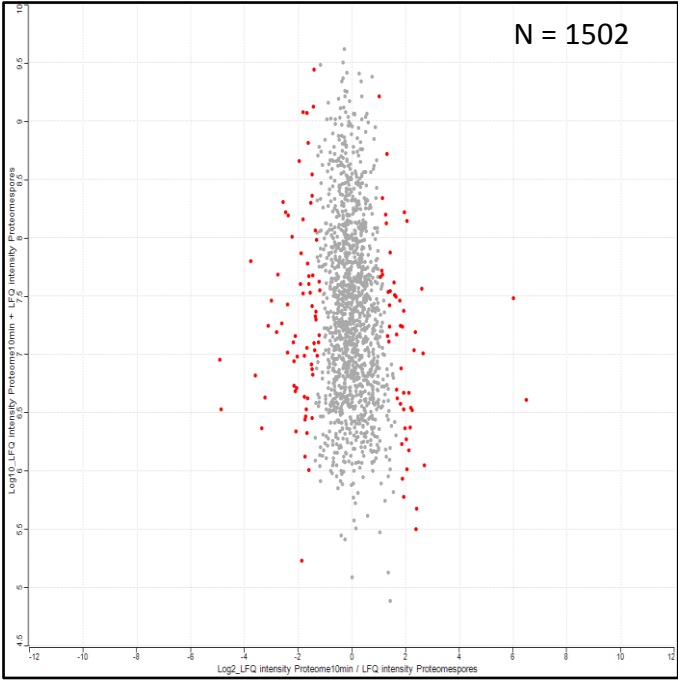

D. 30min (sigB:  $p=0.05$ )

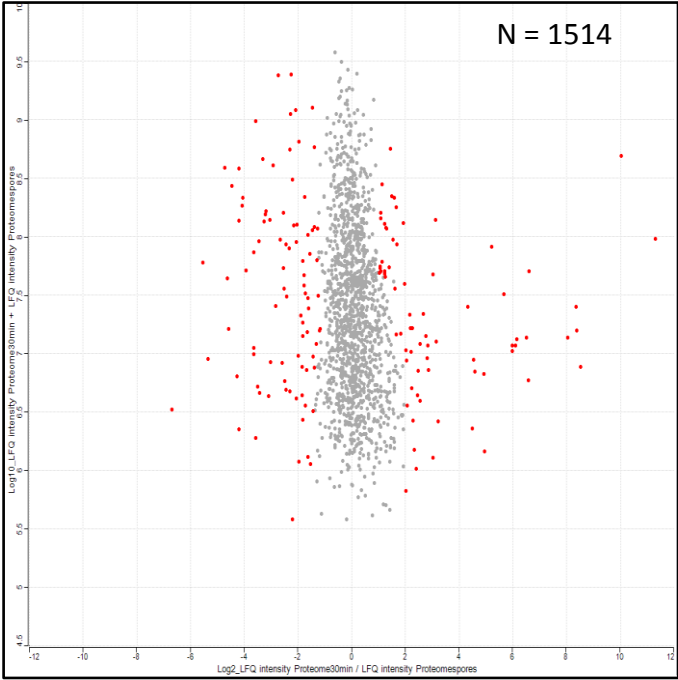

Supplement: Additional file 5: Figure S3. — Overlap in identification of phosphorylated proteins/phospho-sites and distribution of measured protein ratios with assessment of statistical significance. (A) Venn diagram representing the overlap in identification of phosphorylated proteins between the three biological experiments. (B) Venn diagram representing the overlap in identification of phospho-sites between the three biological experiments. (C) Changes in 10 min germinating spore proteome. (D) Changes in 30 min germinating spore proteome. Significantly changing ratios are depicted in red (Significance B, P ≤0.05). (PDF 414 kb) [file 12915_2015_184_MOESM5_ESM.pdf]

Figure S4

A

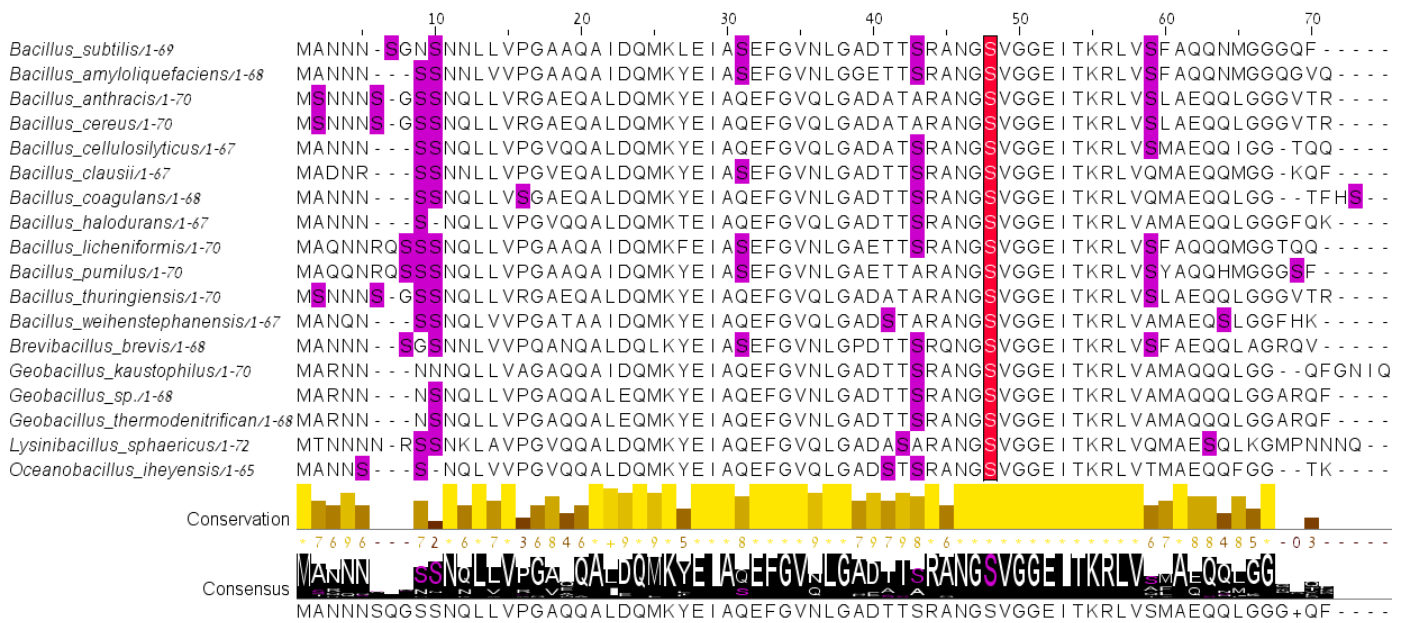

B

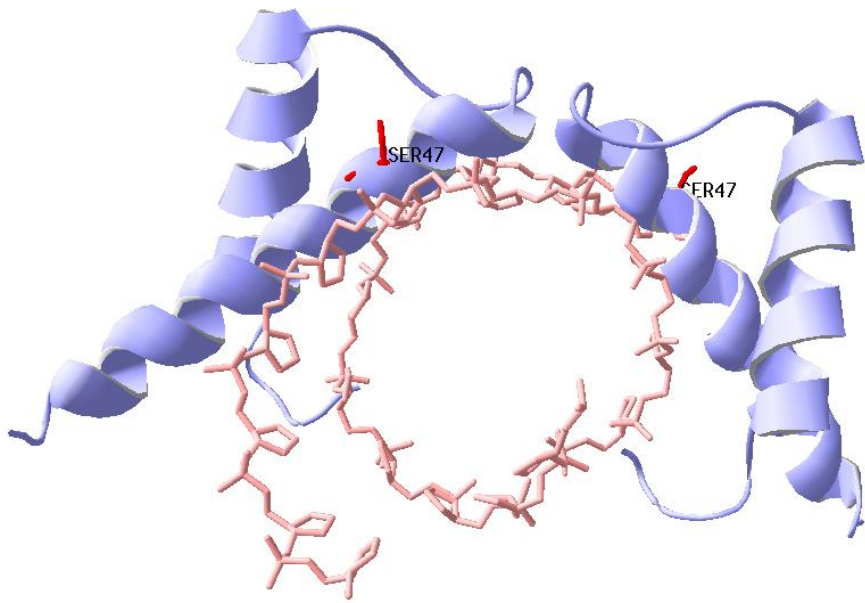

Supplement: Additional file 9: Figure S4. — SspA structure and conservation across Bacillus species. (A) Multiple sequence alignment of B. subtilis SspA and its homologue proteins from representative Bacillus species. Conserved Ser47 residue is highlighted in solid red and other Ser residues are boxed in purple. The corresponding conservation level and consensus sequence are shown below. The multiple sequence alignment was constructed using Jalview. (B) Ribbon diagram of SspA (aa 12–65) protein (cyan) with bound DNA (pink). Ser47 (red) is located at the tip of the second alpha helix. The N and C protein terminals are absent from the structure. Protein structure was predicted by SWISS-MODEL (http://swissmodel.expasy.org/). (PDF 205 kb) [file 12915_2015_184_MOESM9_ESM.pdf]

Figure S5

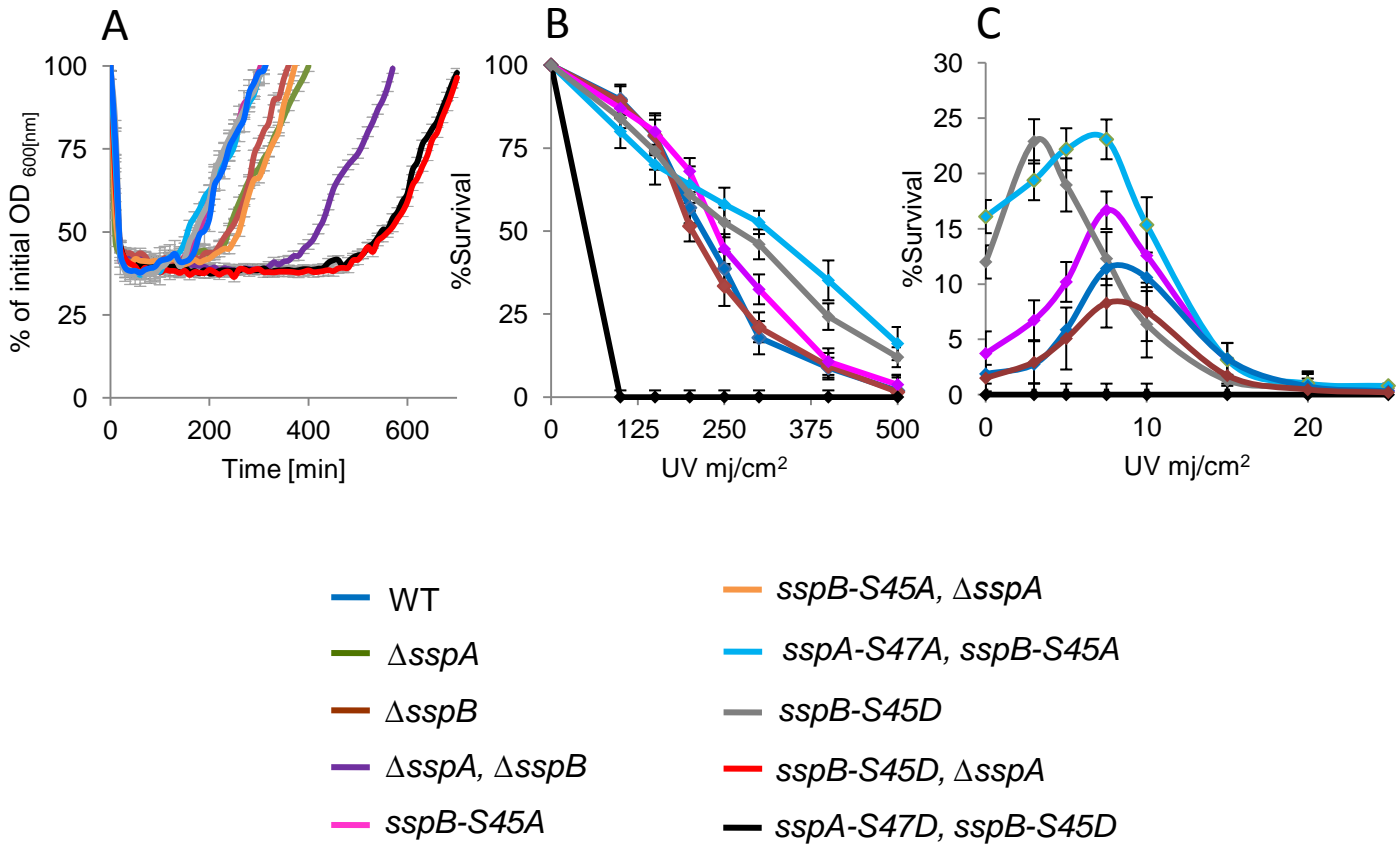

Supplement: Additional file 11: Figure S5. — Characterization of SspB-S45 phosphorylation mutants. (A) Spores of PY79 (WT), AR227 (sspB-S45A), AR228 (sspB-S45D), AR229 (sspA-S47A, sspB-S45A), AR230 (sspA-S47D, sspB-S45D), AR231 (sspB-S45A, ∆sspA), AR232 (sspB-S45D, ∆sspA), AR179 (∆sspA), AR186 (∆sspB), and AR195 (∆sspA, ∆sspB) strains were incubated in S7- supplemented with L-Ala (10 mM) at 37 °C, and optical density (OD600) was measured at the indicated time points. Data are presented as a fraction of the initial OD600 of the phase-bright spores. (B) Spores of PY79 (WT), AR227 (sspB-S45A), AR228 (sspB-S45D), AR229 (sspA-S47A, sspB-S45A), AR230 (sspA-S47D, sspB-S45D), AR231 (sspB-S45A, ∆sspA), AR232 (sspB-S45D, ∆sspA), AR179 (∆sspA), AR186 (∆sspB), and AR195 (∆sspA, ∆sspB) strains were exposed to increasing UV (254 nm) doses (mj/cm2) and plated on LB for viable count. Percentage survival was calculated by dividing the viable spore titer at any UV dose (mj/cm2) with the spore titer obtained from the non-irradiated spores. (C) Spores of PY79 (WT), AR227 (sspB-S45A), AR228 (sspB-S45D), AR229 (sspA-S47A, sspB-S45A), AR230 (sspA-S47D, sspB-S45D), AR231 (sspB-S45A, ∆sspA), AR232 (sspB-S45D, ∆sspA), AR179 (∆sspA), AR186 (∆sspB), and AR195 (∆sspA, ∆sspB) strains were germinated with L-Ala (10 mM). Samples were taken at the indicated time points, irradiated with 500 mj/cm2 UV (254 nm) and plated on LB. Percentage survival was calculated by dividing the viable spore titer at any given time point with the spore titer obtained from spores irradiated at the 0 time point. The data points are averages of results obtained from three independent biological repeats. Error bars designate SD. For simplicity, strains lacking sspA were not depicted in B and C due to their high UV sensitivity (Fig. 3). (PDF 281 kb) [file 12915_2015_184_MOESM11_ESM.pdf]

Figure S6

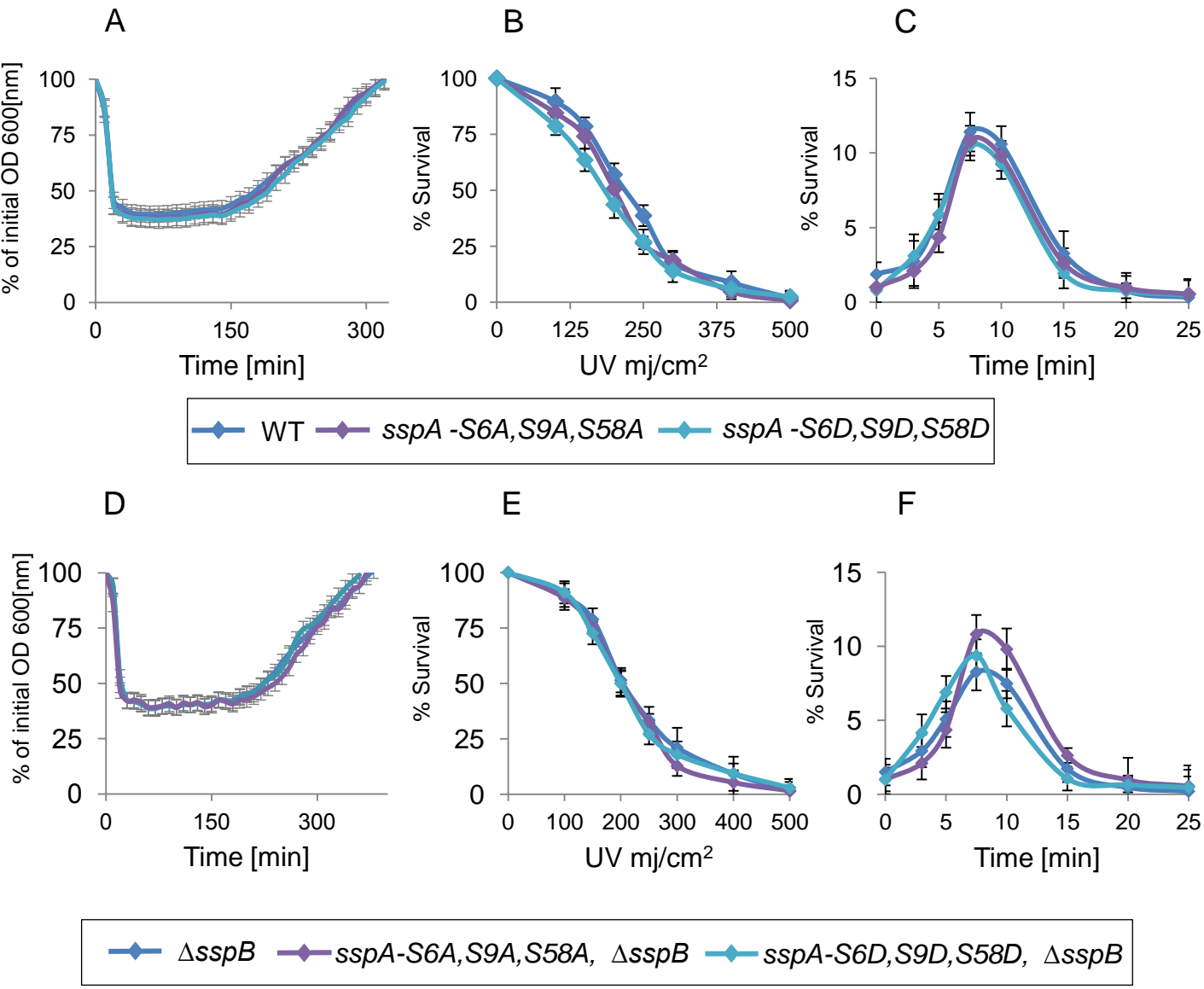

Supplement: Additional file 12: Figure S6. — Characterization of SspA-S6,S9,S58 phosphorylation mutants. (A) Spores of PY79 (wild type, WT), AR211 (sspA-S6A,S9A,S58A), and AR212 (sspA-S6D,S9D,S58D) strains were incubated in S7 defined medium supplemented with L-Ala (10 mM) at 37 °C, and optical density (OD600) was measured at the indicated time points. Data are presented as a fraction of the initial OD600 of the phase-bright spores. Decreasing OD600 signifies spore germination while increasing OD600 indicates spore outgrowth. The data points are averages of results obtained from three independent biological repeats. Error bars designate SD. (B) Spores of PY79 (wild type, WT), AR211 (sspA-S6A,S9A,S58A), and AR212 (sspA-S6D,S9D,S58D) strains were exposed to increasing UV (254 nm) doses (mj/cm2) and plated on LB for viable count. Percentage survival was calculated by dividing the viable spore titer at any given UV dose (mj/cm2) with the spore titer obtained from the non-irradiated spores. The data points are averages of results obtained from three independent biological repeats. Error bars designate SD. (C) Spores of PY79 (wild type, WT), AR211 (sspA-S6A,S9A,S58A), and AR212 (sspA-S6D,S9D,S58D) strains were germinated with L-Ala (10 mM). Samples were taken at the indicated time points, irradiated with 500 mj/cm2 UV (254 nm) and plated on LB. Percentage survival was calculated by dividing the viable spore titer at any given time point with the spore titer obtained from spores irradiated at the 0 time point. The data points are averages of results obtained from three independent biological repeats. Error bars designate SD. (D) Spores of AR186 (∆sspB), AR191 (sspA-S6A,S9A,S58A, ∆sspB), and AR192 (sspA-S6D,S9D,S58D ∆sspB) revival was followed as described in (A). (E) UV resistance of spores of AR186 (∆sspB), AR191 (sspA-S6A,S9A,S58A, ∆sspB), and AR192 (sspA-S6D,S9D,S58D, ∆sspB) was determined as described in (B). (F) UV resistance of AR186 (∆sspB), AR191 (sspA-S6A,S9A,S58A, ∆sspB), and AR192 (sspA S6D,S9D,S58D, ∆sspB [file 12915_2015_184_MOESM12_ESM.pdf]

Figure S7

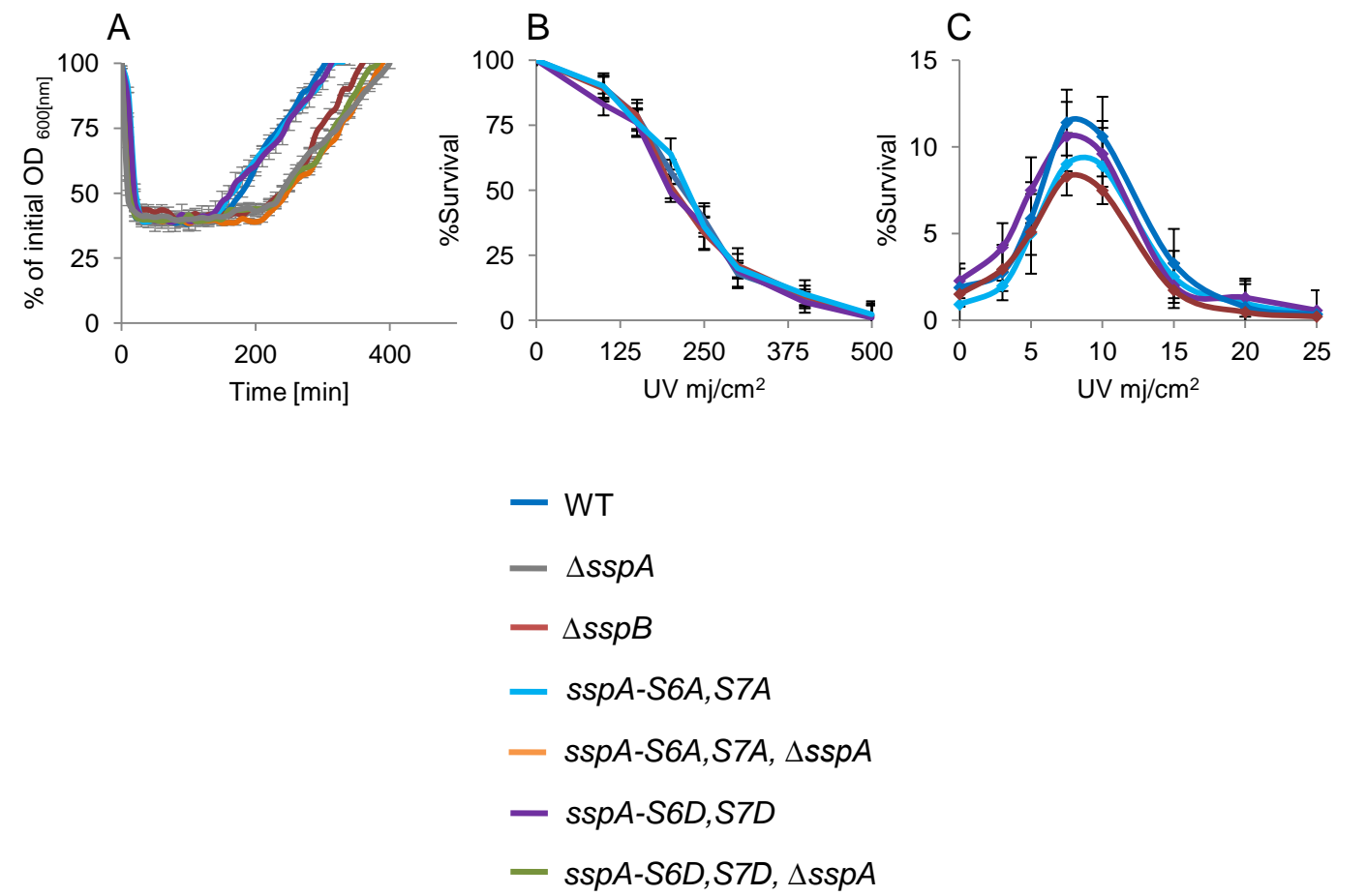

Supplement: Additional file 13: Figure S7. — Characterization of SspB-S6,S7 phosphorylation mutants. (A) Spores of PY79 (wild type, WT), AR233 (sspB-S6A,S7A), AR234 (sspB-S6D,S7D), AR235 (sspB-S6A,S7A, ∆sspA), AR236 (sspB-S6D,S7D, ∆sspA), AR179 (∆sspA), and AR186 (∆sspB) strains were incubated in S7-defined medium supplemented with L-Ala (10 mM) at 37 °C, and optical density (OD600) was measured at the indicated time points. Data are presented as a fraction of the initial OD600 of the phase-bright spores. Decreasing OD600 signifies spore germination while increasing OD600 indicates spore outgrowth. The data points are averages of results obtained from three independent biological repeats. Error bars designate SD. (B) Spores of PY79 (wild type, WT), AR233 (sspB-S6A,S7A), AR234 (sspB-S6D,S7D), AR235 (sspB-S6A,S7A, ∆sspA), AR236 (sspB-S6D,S7D, ∆sspA), AR179 (∆sspA), and AR186 (∆sspB) strains were exposed to increasing UV (254 nm) doses (mj/cm2) and plated on LB for viable count. Percentage survival was calculated by dividing the viable spore titer at any given UV dose (mj/cm2) with the spore titer obtained from the non-irradiated spores. The data points are averages of results obtained from three independent biological repeats. Error bars designate SD. (C) Spores of PY79 (wild type, WT), AR233 (sspB-S6A,S7A), AR234 (sspB-S6D,S7D), AR235 (sspB-S6A,S7A, ∆sspA), AR236 (sspB-S6D,S7D, ∆sspA), AR179 (∆sspA), and AR186 (∆sspB) strains were germinated with L-Ala (10 mM). Samples were taken at the indicated time points, irradiated with 500 mj/cm2 UV (254 nm), and plated on LB. Percentage survival was calculated by dividing the viable spore titer at any given time point with the spore titer obtained from spores irradiated at the 0 time point. The data points are averages of results obtained from three independent biological repeats. Error bars designate SD. For simplicity, strains lacking sspA were not depicted in B and C due to their high UV sensitivity (Fig. 3). (PDF 107 kb) [file 12915_2015_184_MOESM13_ESM.pdf]

Figure S9

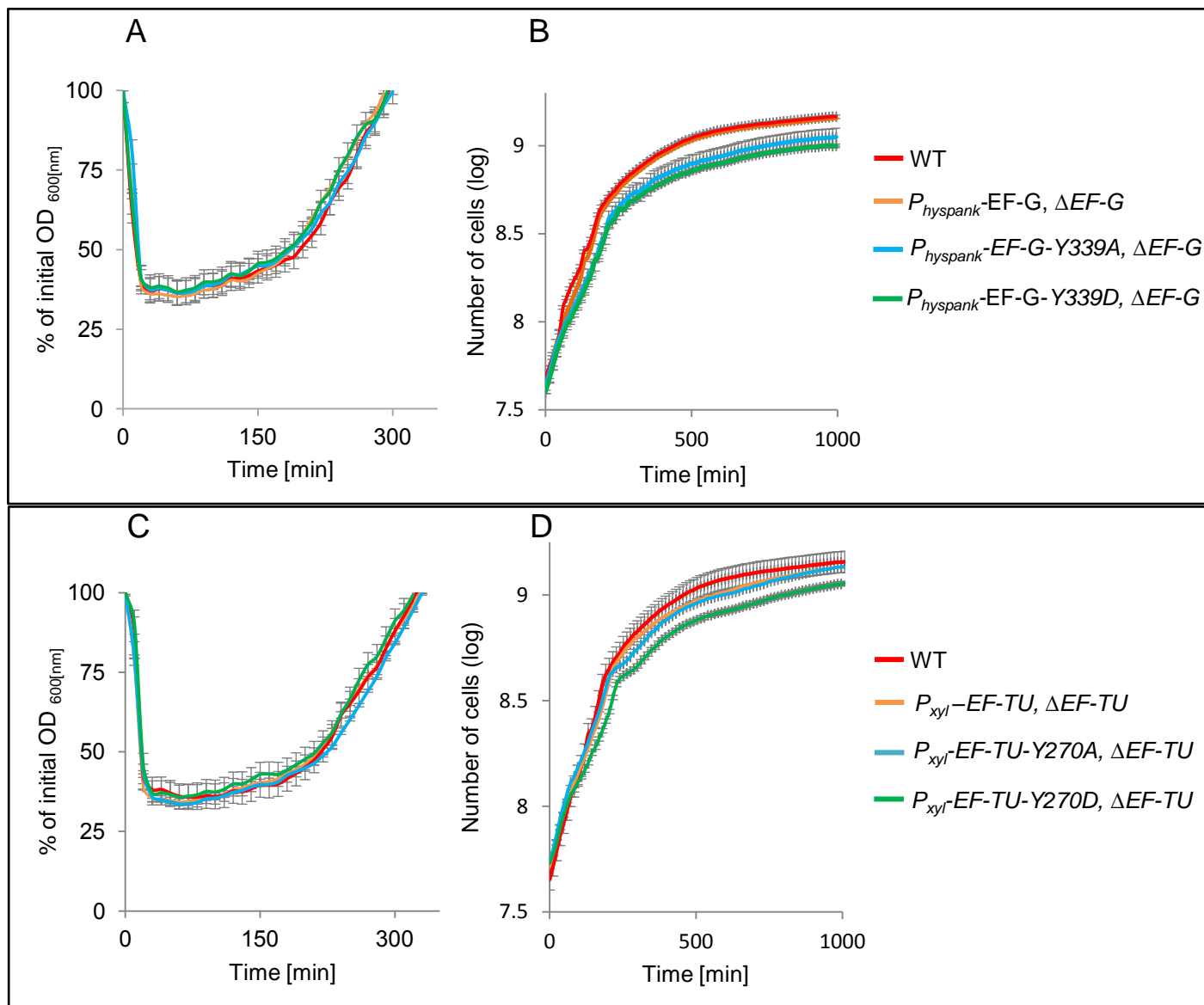

Supplement: Additional file 16: Figure S9. — Phospho-modifications of translation elongation factors affect vegetative growth. (A) Spores of PY79 (WT), AR165 (P hyper-spank -EF-G, ∆EF-G), AR166 (P hyper-spank -EF-G-Y339A, ∆EF-G), and AR167 (P hyper-spank -EF-G-Y339D, ∆EF-G) strains were incubated at 37 °C in S7-defined medium supplemented with L-Ala (10 mM) and 0.5 mM IPTG, and optical density (OD600) was measured at the indicated time points. Data are presented as a fraction of the initial OD600 of the phase-bright spores. Decreasing OD600 signifies spore germination while increasing OD600 indicates spore outgrowth. (B) Strains listed in (A) were grown at 37 °C in S7- supplemented with L-Ala (10 mM) and 0.5 mM IPTG, and OD600 was measured at the indicated time points. AR166 (P hyper-spank -EF-G-Y339A, ∆EF-G) and AR167 (P hyper-spank -EF-G-Y339D, ∆EF-G) strains showed significantly reduced growth rates compared to the control strains by repeated measures ANOVA (P <0.05). C) Spores of PY79 (WT), AR157 (P xyl -EF-TU, ∆EF-TU), AR158 (P xyl -EF-TU-Y270A, ∆EF-TU), and AR159 (P xyl -EF-TU-Y270D, ∆EF-TU) strains were incubated at 37 °C in S7- supplemented with L-Ala (10 mM) and 0.5 % xylose, and OD600 was measured at the indicated time points. Data are presented as a fraction of the initial OD600 of the phase-bright spores. (D) Strains listed in (C) were grown at 37 °C in S7- supplemented with L-Ala (10 mM) and 0.5 % xylose, and OD600 was measured at the indicated time points. The data points are averages of results obtained from three independent biological repeats. Error bars designate SD. AR159 (P xyl -EF-TU-Y270D, ∆EF-TU) showed significantly reduced growth rates compared to the other strains by repeated measures ANOVA (P <0.05). (PDF 237 kb) [file 12915_2015_184_MOESM16_ESM.pdf]

Figure S10

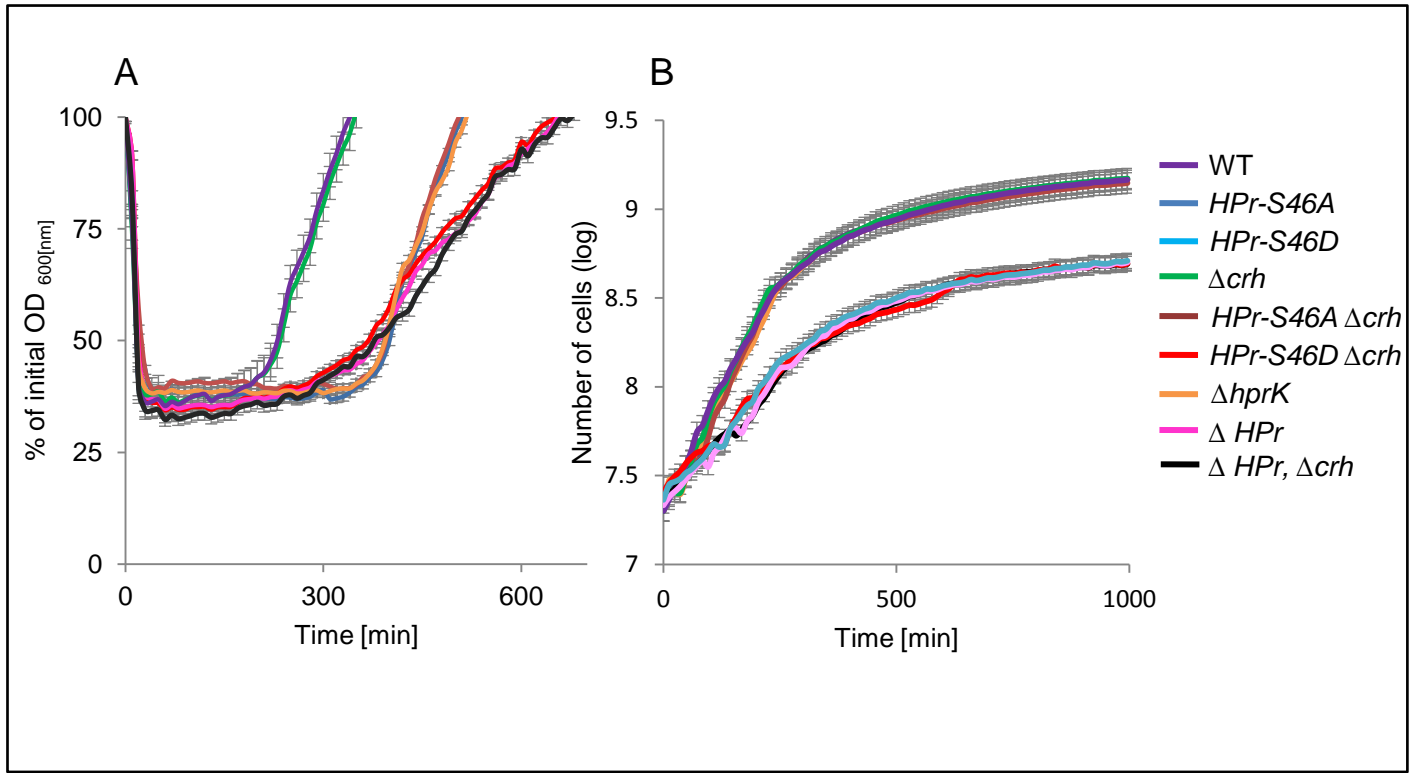

Supplement: Additional file 17: Figure S10. — Analysis of HPr phospho-mutants during spore revival and vegetative growth. (A) PY79 (wild type, WT), AR213 (HPr-S46A), AR214 (HPr-S46D), AR88 (∆crh), AR129 (HPr-S46A, ∆crh), AR130 (HPr-S46D, ∆crh), AR127 (∆HPr), AR128 (∆HPr, ∆crh), and AR196 (∆hprK) strains were incubated at 37 °C in S7-defined medium supplemented with L-Ala (10 mM) and mannose as a sole carbon source, and optical density (OD600) was measured at the indicated time points. Data are presented as a fraction of the initial OD600 of the phase-bright spores. Decreasing OD600 signifies spore germination while increasing OD600 indicates spore outgrowth. The data points are averages of results obtained from three independent biological repeats. Error bars designate SD. (B) Growth curves of PY79 (wild type, WT), AR213 (HPr-S46A), AR214 (HPr-S46D), AR88 (∆crh), AR129 (HPr-S46A, ∆crh), AR130 (HPr-S46D, ∆crh), AR127 (∆HPr), AR128 (∆HPr, ∆crh), and AR196 (∆hprK) strains. Cells were grown at 37 °C in S7 medium supplemented with L-Ala (10 mM) and mannose as a sole carbon source, and OD600 was measured at the indicated time points. The data points are averages of results obtained from three independent biological repeats. Error bars designate SD. (PDF 233 kb) [file 12915_2015_184_MOESM17_ESM.pdf]

Figure S11

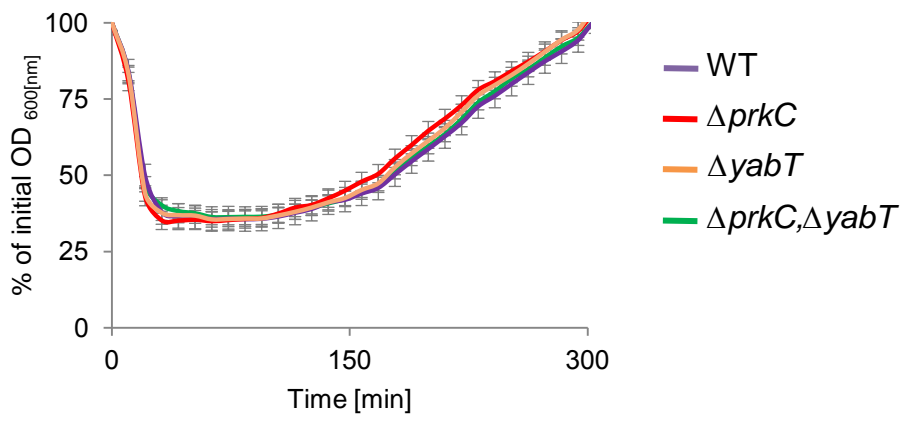

Supplement: Additional file 18: Figure S11. — The absence of identified kinases does not affect spore revival. Spores of PY79 (wild type, WT), AR73 (∆prkC), AR102 (∆yabT), and AR114 (∆prkC, ∆yabT), strains were incubated at 37 °C in S7-defined medium supplemented with L-Ala (10 mM), and optical density (OD600) was measured at the indicated time points. Data are presented as a fraction of the initial OD600 of the phase-bright spores. Decreasing OD600 signifies spore germination while increasing OD600 indicates spore outgrowth. The data points are averages of results obtained from four independent biological repeats. Error bars designate SD. (PDF 100 kb) [file 12915_2015_184_MOESM18_ESM.pdf]
