# Supplementary material for: Phosphoproteome dynamics mediate revival of bacterial spores
Source: BMC Biol. 2015 Sep 17;13:76. doi: 10.1186/s12915-015-0184-7 (PMC4574613; doi:10.1186/s12915-015-0184-7)
Supplement: Additional file 3: Figure S2. — Comparison of germinating spore and vegetative growth phosphoproteomes. (A) Overlap between the germinating and vegetative phosphoproteins. (B) Common phosphorylation sites between the germinating and vegetative phase phosphoproteins. (C) The identity of the overlapping 17 phosphorylation sites and the stage of vegetative growth at which they show increased phosphorylation. (PDF 185 kb) [file 12915_2015_184_MOESM3_ESM.pdf]

Figure S2

A. Phosphoproteins

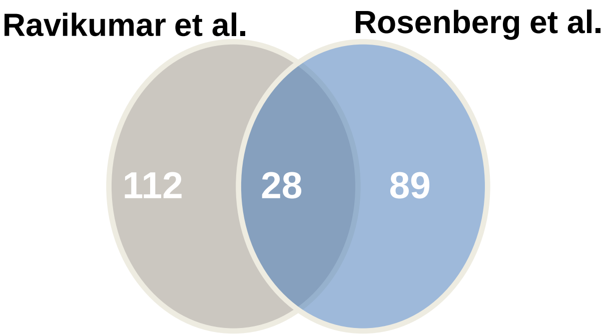

B. Phosphorylation sites

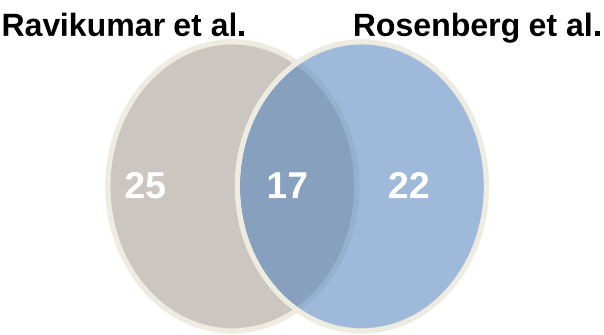

C.

Protein name	Protein IDs	Position	Stage (Peak)
azoR2	O32224	S75	T5
HPr	P08877	S46	T5
HPr	P08877	S12	T5
RpsH	P12879	S55	T1,T3
RsbV	P17903	S56	T4,T5
icd	P39126	S168	T5,T3
FabI	P54616	S203	T5
GreA	P80240	S84	T3,T5
SucC	P80886	S220	T5
ValS	Q05873	S529	T5
PrkC	O34507	T290	T1
RsbRB	O34860	T186	T3
RplN	P12875	T6	T4
FbaA	P13243	T234	T5,T4
YabS	P37561	T88	T5
YabS	P37561	T90	T5
SodA	P54375	T47	T1,T5

T1) Exponential growth
T2) Entry into retardation phase
T3) Transition to stationary phase
T4) Early stationary phase
T5) Late stationary phase
