# Supplementary material for: Phosphoproteome dynamics mediate revival of bacterial spores
Source: BMC Biol. 2015 Sep 17;13:76. doi: 10.1186/s12915-015-0184-7 (PMC4574613; doi:10.1186/s12915-015-0184-7)
Supplement: Additional file 8: Table S5. — Deletion- and site-directed mutants assessed in this study. (PDF 206 kb) [file 12915_2015_184_MOESM8_ESM.pdf]

Table S5: Deletion and site directed mutants assessed in this study

**Gene deletion strains**		
**Biological process**	**Mutant strain**	**Revival Phenotype (assayed by monitoring change in OD_{600})***
**Sporulation**	Δ*sspA*	outgrowth delay in minimal media
	Δ*sspB*	outgrowth delay in minimal media
	Δ*sspA*, Δ*sspB*	outgrowth delay in minimal media
	Δ*floT*	
	Δ*floA*	
	Δ*floT*, Δ*floA*	
	Δ*swrC*	
**Carbon Metabolism**	Δ*HPr*	outgrowth delay
	Δ*crh*	no effect
	Δ*hprK*	outgrowth delay
	Δ*fruA*	no effect
**Transcription**	Δ*rpoE*	no effect
	Δ*phoRD*	no effect
**Stress response**	Δ*rsbRB*	no effect
	Δ*rsbRC*	no effect
	Δ*rsbRD*	no effect
	Δ*RsbRB*, Δ*RsbRC*, Δ*RsbRD*	no effect
	Δ*sigB*	outgrowth delay under stress conditions
	Δ*bmrU*	no effect
**Ser/Thr Kinases**	Δ*prkC*	no effect
	Δ*yabT*	no effect
	Δ*yabT*,Δ*prkC*	no effect
**Point mutant strains**		
**Sporulation**	*sspA-S47A*	no effect
	sspA-S47D	no effect
	sspA-S47A, Δ*sspB*	no effect
	sspA-S47D, Δ*sspB*	outgrowth delay in rich and poor media
	sspA-S6A,S9A,S58A	no effect
	sspA-S6D,S9D,S58D	no effect

	sspA-S6A,S9A,S58A, ΔsspB	no effect
	sspA-S6D,S9D,S58D, ΔsspB	no effect
	sspB-S45A	no effect
	sspB-S45D	no effect
	sspA-S47A, sspB-S45A	no effect
	sspA-S47D, sspB-S45D	outgrowth delay in rich and poor media
	sspB-S45A, ΔsspA	no effect
	sspB-S45D, ΔsspA	outgrowth delay in rich and poor media
	sspB S6A,S7A,	no effect
	sspB S6D,S7D	no effect
	sspB S6A,S7A, ΔsspA	no effect
	sspB S6D,S7D, ΔsspA	no effect
	floT-S112A	no effect
	floT-S112D	no effect
	floT-S112A, ΔfloA	no effect
	floT-S112D, ΔfloA	no effect
**Translation**	*rpsJ-S32A*	no effect
	rpsJ-S32D	outgrowth delay
	EF-G -Y339A	no effect
	EF-G -Y339D	no effect
	EF-TU-Y270A	no effect
	EF-TU-Y270D	no effect
**Carbon metabolism**	*HPr-S46A*	outgrowth delay
	HPr-S46D	outgrowth delay
	HPr-S46A, Δcrh	outgrowth delay
	HPr-S46D, Δcrh	outgrowth delay
	HPr-S12A	no effect
	HPr-S12D	no effect
	HPr-S12A, Δcrh	no effect
	HPr-S12D, Δcrh	no effect
**Stress response**	*bmrU Y162A*	no effect

	bmrU Y162D	no effect
	rsbV S56D	outgrowth delay under stress conditions
**Ser/Thr Kinases**	Δ*prkC S214A,S217A*	no effect
	Δ*prkCS214D,S217D*	no effect

*For each mutant revival was induced by L-Ala and AGFK separately.
