# Supplementary material for: Phosphoproteome dynamics mediate revival of bacterial spores
Source: BMC Biol. 2015 Sep 17;13:76. doi: 10.1186/s12915-015-0184-7 (PMC4574613; doi:10.1186/s12915-015-0184-7)
Supplement: Additional file 10: Table S6. — Sporulation efficiency of strains used in this study. (PDF 102 kb) [file 12915_2015_184_MOESM10_ESM.pdf]

Table S6: Sporulation efficiency of strains used in this study

Strain	Genotype	Sporulation efficiency %
PY79	Wild type	100
AR209	*sspA S47A*	95.3±8.2
AR210	*sspA S47D*	85.4±11.3
AR187	*sspA S47A, sspB::spc*	94.5±8.7
AR188	*sspA S47D, sspB::spc*	25.1±7.1
AR211	*sspA S6A, S9A, S58A*	101±4.8
AR212	*sspA S6D, S9D, S58D*	97.96±3.1
AR191	*sspA S6A, S9A, S58A, sspB::spc*	93.1±6.2
AR192	*sspA S6D, S9D, S58D, sspB::spc*	98.7±5.6
AR227	*sspB-S45A*	96.5±2.8
AR228	*sspB-S45D*	91.5±5.6
AR229	*sspA-S47A, sspB-S45A*	93.9±4.1
AR230	*sspA-S47D, sspB-S45D*	29.9±5.9
AR231	*sspB-S45A, sspA::kan*	92.7±6.2
AR232	*sspB-S45D, sspA::kan*	19.4±4.8
AR233	*sspB S6A,S7A*	97.4±3.9
AR234	*sspB S6D,S7D*	92.7±8.1
AR235	*sspB S6A,S7A, sspA::kan*	94.8±5.8
AR236	*sspB S6D,S7D, sspA::kan*	96.2±3.6
AR237	*sspA::kan*	95.8±4.7
AR186	*sspB::spc*	94.9±6.8

AR179	*sspA::mls*	93.5±7.1
AR195	*sspA::mls, sspB::spc*	95.8±5.1
AR168	*amyE::*P_{xyl}*- rpsJ-spc, rpsJ:cat*	91.8±7.5
AR169	*amyE::*P_{xyl}*-rpsJS32A-spc, rpsJ::cat*	99.4±4.0
AR185	*amyE::*P_{xyl}*-rpsJ S32D-spc, rpsJ::cat*	110.6±4.9
AR165	*amyE::*$P_{hyper\text{-}spank}$*-EF-G-spc, EF-G::cat*	92.3±8.5
AR166	*amyE::*$P_{hyper\text{-}spank}$*-EF-G Y339A-spc, EF-G::cat*	97.9±4.4
AR167	*amyE::*$P_{hyper\text{-}spank}$*-EF-G Y339D spc, EF-G::cat*	103.4±7.9
AR157	*amyE::*$P_{hyper\text{-}spank}$*- EF-TU Y270, EF-TU::cat*	97.2±6.7
AR158	*amyE::*$P_{hyper\text{-}spank}$*- EF-TU Y270*A*, EF-TU::cat*	98.4±3.7
AR159	*amyE::*$P_{hyper\text{-}spank}$*- Y270D, EF-TU::cat*	92.7±7.4
AR213	*Hpr S46A*	10.4±3.2
AR214	*Hpr S46D*	50.5±5.8
AR129	*Hpr S46A,* c*rh:spc*	6.7±2.3
AR130	*Hpr S46D, crh:spc*	47.2±4.9
AR127	*Hpr::cat*	51.7±4.7
AR128	*Hpr::cat, crh::spc*	41.4±3.1
AR88	*crh::spc*	96.8±5.8
AR196	*hprK::spc*	17.4±6.3
AR73	*prkC::kan*	85.2±5.1
AR102	*yabT::tet*	89.7±5.8
AR114	*yabT::tet, prkC:kan*	78.6±6.9
