# Supplementary material for: Phosphoproteome dynamics mediate revival of bacterial spores
Source: BMC Biol. 2015 Sep 17;13:76. doi: 10.1186/s12915-015-0184-7 (PMC4574613; doi:10.1186/s12915-015-0184-7)
Supplement: Additional file 19. — Supporting Information. Supporting Methods, Supporting Tables S8–S10. (PDF 366 kb) [file 12915_2015_184_MOESM19_ESM.pdf]

Additional file 19 - Supporting Information

Phosphoproteome Dynamics Mediate Revival of Bacterial Spores

Rosenberg et al.

Supporting Information Contains:

Supporting Methods

Supporting Tables (S8-S10)

Supporting Methods

Strain construction

All strains used in this study are derivative of PY79 [1] and are listed in Table S8. All plasmids and primers used in this study are listed in Table S9 and S10, respectively. Allele insertion deletion was generated by long flanking homology PCR as described previously [2].

Allele insertion deletion

(AR186) *sspB::spc*- The Δ*sspB::spc* insertion deletion allele was generated by long flanking homology PCR using primers 1349 and1350, 1351 and 1352 (Table S10).

(AR179) *sspB::mls*- The Δ*sspB::mls* insertion deletion allele was generated by long flanking homology PCR using primers 2505 and1350, 2506 and 2507 (Table S10).

(AR238) *sspA::kan*- The Δ*sspB::mls* insertion deletion allele was generated by long flanking homology PCR using primers 1345 and1346, 1347 and 1348 (Table S10).

(AR179) *sspA::mls*- The Δ*sspA::mls* insertion deletion allele was generated by long flanking homology PCR using primers 2420 and2421, 2422 and 2423 (Table S10).

(AR195) *sspB::spc, sspA::mls*- The *sspB::spc, sspA::mls* insertion deletion alleles were generated by transformation of AR179 with genomic DNA from AR186.

(AR88) *crh::spc*- The Δ*crh::spc* insertion deletion allele was generated by long flanking homology PCR using primers 1474 and 1475, 1476 and 1477 (Table S10).

(AR127) *HPr::cat*- The Δ*Hpr::*cat insertion deletion allele was generated by transformation of PY79 with genomic DNA from MZ303[3].

(AR128) *HPr::cat, crh::spc*- The *HPr::cat,* Δ*crh::spc* insertion deletion allele was generated by transformation of AR127 with genomic DNA from AR88.

(AR196) *hprK::spc*- The Δ*hprK::*spc insertion deletion allele was generated by transformation of PY79 with genomic DNA from GP202 [4].

(AR73) *prkC::kan*- The Δ*prkC:kan* insertion deletion allele was generated by long flanking homology PCR using primers 1466 and 1467, 1468 and 1469 (Table S10).

(AR102) *yabT::tet*- The Δ*yabT::tet* insertion deletion allele was generated by long flanking homology PCR using primers 2022 and 2023, 2024 and 2025 (Table S10).

(AR114) *yabT::tet*, *prkC:kan*- The Δ*yabT::tet*, Δ*prkC:kan* insertion deletion alleles were generated by transformation of AR73 with genomic DNA from AR102.

Construction of point mutant strains

(AR209) *sspA-S47A*- To replace the wild type allele of *sspA* with the *sspA-S47A* allele, a PCR product containing ~500 bp upstream and ~500 bp downstream of the *sspA-S47* mutation was amplified using chromosomal DNA purified from PY79 as the template and primer pair 1403/1404. The PCR product was digested with *BamH*I and *Sal*I, and cloned into the *BamH*I and *Sal*I sites of pMINImad2 to generate pAR200. Site directed mutagenesis was conducted on pAR200 to change the codon encoding Ser47 to a codon encoding Ala using primer pair 2297/2298 and the Quickchange II kit (Stratagene) to create pAR201. The plasmid pAR201 was introduced into PY79 by single crossover integration by transformation at the restrictive temperature for plasmid replication (37ºC), using *mls* resistance as a selective marker. To evict the plasmid, the strain was incubated in 3 ml LB at a permissive temperature for plasmid replication (23ºC) for 14 hrs, diluted 30 fold into fresh LB, and incubated at 23ºC for another 8 hrs. Cells were then serially diluted and plated on LB agar at 37ºC. Individual colonies were patched on LB plates and LB plates containing *mls*, to identify *mls* sensitive colonies that had

evicted the plasmid. Chromosomal DNA from colonies that had excised the plasmid was purified and screened by PCR using primers 1403/1404 to determine which isolate had retained the *sspA-S47A* allele (e.g. AR209).

(AR210) *sspA-S47D*- Site directed mutagenesis was conducted on pAR200 to change the codon encoding Ser47 to a codon encoding Asp using primer pair 2299/2300 and the Quickchange II kit (Stratagene) to create pAR202. The plasmid pAR202 was introduced into PY79 and the mutant selected as described for AR209.

(AR187) *sspA-S47A sspB::spc*- The insertion deletion allele was generated by transformation of AR209 with genomic DNA from AR186.

(AR188) *sspA-S47D sspB::spc*- The insertion deletion allele was generated by transformation of AR210 with genomic DNA from AR186.

(AR211) *sspA-S6A,S9A,S58A*- Sequential site directed mutagenesis was conducted on pAR200 to change the codons encoding Ser6,9 and 58 to a codons encoding Ala using primer pairs 1405/1406, 1829/1830, 1641/1642, respectively, and the Quickchange II kit (Stratagene) to create pAR211. The plasmid pAR211 was introduced into PY79 and the mutant was selected as described for AR209.

(AR212) *sspA-S6D,S9D,S58D*- Sequential site directed mutagenesis was conducted on pAR200 to change the codons encoding Ser6,9 and 58 to a codon encoding Asp using primer pairs 1407/1408, 1831/1832, 1643/1644, respectively, and the Quickchange II kit (Stratagene) to create pAR212. The plasmid pAR212 was introduced into PY79 and the mutant was selected as described for AR209.

(AR191) *sspA-S6A,S9A,S58A,sspB::spc*- The insertion deletion allele was generated by transformation of AR191 with genomic DNA from AR186.

(AR192) *sspA-S6D,S9D,S58D,sspB::spc*- The insertion deletion allele was generated by transformation of AR192 with genomic DNA from AR186.

(AR200) *sspA-gfp*$_{A206K}$*-spc, sspB::mls*-To create a translational fusion - *sspA-gfp*$_{A206K}$, a PCR product containing the 3' region of *sspA* was amplified using chromosomal DNA purified from AR210 as the template and primer pair 1941/1942. The PCR product was digested with *Mfe*I and *Xho*I, and cloned into the *EcoRI*I and *Xho*I sites of pAR100 which contains the *gfp*$_{A206K}$ coding sequence to generate pAR230. The plasmid pAR230 was introduced into AR179 by single crossover integration.

(AR206) *sspA-S47D-gfp*$_{A206K}$*-spc, sspB::mls*- To create a translational fusion - *sspAS47D-gfp*$_{A206K}$ a PCR product containing the 3' region of *sspA-S47D* was amplified using chromosomal DNA purified from PY79 as the template and primer pair 1941/1942. The PCR product was digested with *Mfe*I and *Xho*I, and cloned into the *EcoRI*I and *Xho*I sites of pAR100 which contains the *gfp*$_{A206K}$ coding sequence to generate pAR229. The plasmid pAR229 was introduced into AR179 by single crossover integration.

(AR227) *sspB-S45A*- To replace the wild type allele of *sspB* with the *sspB-S45A* allele, a PCR product containing ~500 bp upstream and ~500 bp downstream of the *sspB-S45* mutation was amplified using chromosomal DNA purified from PY79 as the template and primer pair 2900/2901. The PCR product was digested with *KpnI* and *Sal*I, and cloned into the *Kpn*I and *Sal*I sites of pMINImad2 to generate pAR241. Site directed mutagenesis was conducted on pAR241 to change the codon encoding Ser45 to a codon encoding Ala using primer pair 2896/2897 and the Quickchange II kit (Stratagene) to create pAR242. The plasmid pAR242 was introduced into PY79 and the mutant was selected as described for AR209.

(AR228) *sspB-S45D* Site directed mutagenesis was conducted on pAR241 to change the codon encoding Ser45 to a codon encoding Asp using primer pair 2898/2899 and the Quickchange II kit (Stratagene) to create pAR243. The plasmid pAR243 was introduced into PY79 and the mutant selected as described for AR209.

(AR229) *sspA-S47A sspB-S45A* The plasmid pAR242 was introduced into AR209 and the mutant selected as described for AR209.

(AR230) *sspA-S47D sspB-S45D* The plasmid pAR243 was introduced into AR210 and the mutant selected as described for AR209.

(AR231) *sspB-S45A ΔsspA* The insertion deletion allele was generated by transformation of AR227 with genomic DNA from AR237.

(AR232) *sspB-S45D ΔsspA* The insertion deletion allele was generated by transformation of AR228 with genomic DNA from AR237.

(AR233) *sspB-S6A,S7A* Site directed mutagenesis was conducted on pAR241 to change the codons encoding Ser6 and Ser7 to codons encoding Ala using primer pair 2892/2893 and the Quickchange II kit (Stratagene) to create pAR244. The plasmid pAR244 was introduced into PY79 and the mutant selected as described for AR209.

(AR234) *sspB-S6D,S7D* Site directed mutagenesis was conducted on pAR241 to change the codons encoding Ser6 and Ser7 to codons encoding Asp using primer pair 2894/2895 and the Quickchange II kit (Stratagene) to create pAR245. The plasmid pAR245 was introduced into PY79 and the mutant selected as described for AR209.

(AR235) *sspB-S6A,S7A ΔsspA* The insertion deletion allele was generated by transformation of AR233 with genomic DNA from AR237.

(AR236) *sspB-S6D,S7D ΔsspA* The insertion deletion allele was generated by transformation of AR234 with genomic DNA from AR237.

(AR213) *HPr-S46A*- To replace the wild type allele of *HPr* with the *HPr-S46A* allele, a PCR product containing ~500 bp upstream and ~500 bp downstream of the *HPrS46* mutation was amplified using chromosomal DNA purified from PY79 as the template and primer pair 1246/1247, digested with *BamH*I and *Sal*I, and cloned into the *BamH*I and *Sal*I sites of pMINImad2 [5] to generate pAR213. Site directed mutagenesis was conducted on pAR213 to change the codon encoding Ser46 to a codon encoding Ala using primer pair 2291/2292 and the Quickchange II kit (Stratagene) to create pAR214. The plasmid pAR214 was introduced into PY79 and the mutant was selected as described for AR209.

(AR214) *HPr-S46D*- Site directed mutagenesis was conducted on pAR213 to change the codon encoding Ser46 to a codon encoding Asp using primer pair 2293/2294 and the Quickchange II kit (Stratagene) to create pAR215. The plasmid pAR215 was introduced into PY79 and the mutant was selected as described for AR209.

(AR129) *HPr-S46A, crh:spc*- The insertion deletion allele was generated by transformation of AR213 with genomic DNA from AR88.

(AR130) *HPr-S46D, crh:spc*- The insertion deletion allele was generated by transformation of AR214 with genomic DNA from AR88.

(AR168) *amyE::P$_{xyl}$-rpsJ-spc, rpsJ::cat*- To generate the inducible *amyE::Pxyl-rpsJ-spc* construct pAR218, a PCR product containing *rpsJ* was amplified from PY79 chromosomal DNA using the primer pair 2235/2236, digested with *Sal*I and *BamH*I and cloned into the *Sal*I and *BamH*I sites of pDR150 containing a spectinomycin resistance cassette, a polylinker downstream of the *Pxyl* promoter, and the gene encoding the XylR repressor between the arms of the *amyE*

gene. The linearised plasmid pAR218 was introduced into PY79 by double crossover integration, producing the strain AR122. Then, *rpsJ* allele at the native site was replaced with promoterless *cat* allele by long flanking homology PCR using primers 2403 and 2404, 2405 and 2406 (Table S10).

(AR169) *amyE* P_{xyl}*-rpsJ-S32A-spc, rpsJ::cat*- To generate the inducible *amyE::Pxyl-rpsJ-S32A-spc* construct pAR219, site directed mutagenesis was conducted on pAR218 using primer pair 2287/2288 and the Quickchange II kit (Stratagene). The linearised plasmid pAR219 was introduced into PY79 by double crossover integration producing the strain AR123. Then *rpsJ* allele at the native site was replaced with promoterless *cat* allele by long flanking homology PCR using primers 2403 and 2404, 2405 and 2406 (Table S10).

(AR185) *amyE* P_{xyl}*-rpsJ-S32D-spc, rpsJ::cat*- To generate the inducible *amyE::Pxyl-rpsJS32D-spc* construct pAR220, site directed mutagenesis was conducted on pAR218 using primer pair 2289/2290 and the Quickchange II kit (Stratagene). The linearised plasmid pAR220 was introduced into PY79 by double cross-over integration producing the strain AR124. Then *rpsJ* allele at the native site was replaced with promoterless *cat* allele by long flanking homology PCR using primers 2403 and 2404, 2405 and 2406 (Table S10).

(AR223) *amyE::Pxyl- rpsJ-HA-spc-kan, rpsJ::cat*- To generate the inducible *amyE::Pxyl-rpsJ-HA-spc-kan* starin a PCR product of the 3' region downstream to the S32 codon of *rpsJ* was amplified using chromosomal DNA purified from PY79 as the template and primer pair 2704/2705 (with 2707 containing the HA-tag coding sequence) . The PCR product was digested with *Sac*I and *BamH*I, and cloned into the *Sac*I and *BamH*I sites of pDG780 a *B. subtilis* suicide integration vector to generate pAR240. The plasmid pAR240 was introduced into AR168 by single crossover integration in the presence of 0.5% xyl.

(AR224) *amyE::Pxyl-rpsJ-S32A-HA-spc-kan, rpsJ::cat*- To generate the inducible *amyE::Pxyl-rpsJ-S32A-HA-spc-kan, rpsJ::cat* strain, the plasmid pAR240 was introduced into AR169 by single crossover integration in the presence of 0.5% xyl.

(AR225) *amyE P_{xyl}-rpsJ-S32D-spc, rpsJ::cat*- To generate the inducible *amyE::Pxyl-rpsJ-HA-spc-kan* strain the plasmid pAR240 was introduced into AR185 by single crossover integration in the presence of 0.5% xyl.

(AR165) *amyE::$P_{hyper-spank}$-EF-G-spc, EF-G::cat*- To generate the inducible *amyE::$P_{hyper-spank}$-EF-G* construct pAR221, a PCR product containing *EF-G* was amplified from PY79 chromosomal DNA using the primer pair 1775/2169, digested with *Hind*III and *Sph*I and cloned into the *Hind*III and *Sph*I sites of pDR111, containing a spectinomycin resistance cassette, a polylinker downstream of the *Phyperspank* promoter, and the gene encoding the *lacI* repressor between the arms of the *amyE* gene. The linearised plasmid pAR221 was introduced into PY79 by double crossover integration producing the strain AR121. Then, *EF-G* allele at the native site was replaced with promoterless *cat* allele by long flanking homology PCR using primers 2407 and 2408, 2409 and 2410 (Table S10).

(AR166) *amyE::$P_{hyper-spank}$-EF-G-Y339A-spc, EF-G::cat*- To generate the *amyE::$P_{hyper-spank}$-EF-G-Y339A-spc* construct pAR222, site directed mutagenesis was conducted on pAR221 using primer pair 1462/1463 and the Quickchange II kit (Stratagene). The linearised plasmid pAR222 was introduced into PY79 by double crossover integration producing the strain AR90. Then *EF-G* allele at the native site was replaced with promoterless *cat* allele by long flanking homology PCR using primers 2407 and 2408, 2409 and 2410 (Table S10).

(AR167) *amyE::$P_{hyper-spank}$-EF-G-Y339D spc, EF-G::cat*- To generate the *amyE::$P_{hyper-spank}$-EF-G-Y339D-spc* construct pAR223, site directed mutagenesis was conducted on pAR221 using

primer pair 1464/1465 and the Quickchange II kit (Stratagene). The linearised plasmid pAR223 was introduced into PY79 by double crossover integration producing the strain AR91. Then *EF-G* allele at the native site was replaced with promoterless *cat* allele by long flanking homology PCR using primers 2407 and 2408, 2409 and 2410 (Table S10).

(AR157) *amyE::*P_{xyl}*-EF-TU, EF-TU::cat*- To generate the inducible *amyE::Pxyl-EF-TU-spc* construct pAR224, a PCR product containing *EF-TU* was amplified from PY79 chromosomal DNA using the primer pair 2233/2234, digested with *Sal*I and *BamH*I and cloned into the *Sal*I and *BamH*I sites of pDR150 containing a spectinomycin resistance cassette, a polylinker downstream of the *Pxyl* promoter, and the gene encoding the XylR repressor between the arms of the *amyE* gene. The linearised plasmid pAR224 was introduced into PY79 by double crossover integration producing the strain AR146. Then, *EF-TU* allele at the native site was replaced with promoterless *cat* allele by long flanking homology PCR using primers 2385 and 2386, 2387 and 2388 (Table S10).

(AR158) *amyE::*P_{xyl}*-EF-TU-Y270*A, *EF-TU::cat*- To generate the inducible *amyE::Pxyl- EF-TU Y270*A-*spc* construct pAR225, site directed mutagenesis was conducted on pAR224 using primer pair 1778/1779 and the Quickchange II kit (Stratagene). The linearised plasmid pAR225 was introduced into PY79 by double crossover integration producing the strain AR147. Then *EF-TU* allele at the native site was replaced with promoterless *cat* allele by long flanking homology PCR using primers 2385 and 2386, 2387 and 2388 (Table S10).

(AR159) *amyE::*$P_{hyper\text{-}spank}$*-EF-TU-Y270D, EF-TU::cat*- To generate the inducible *amyE::Pxyl-EF-TU Y270*D-*spc* construct pAR226, site directed mutagenesis was conducted on pAR224 using primer pair 1780/1781 and the Quickchange II kit (Stratagene). The linearised plasmid pAR226 was introduced into PY79 by double crossover integration producing the strain AR148.

Then, *EF-TU* allele at the native site was replaced with promoterless *cat* allele by long flanking homology PCR using primers 2385 and 2386, 2387 and 2388 (Table S10).

(AR217) *rsbV-S56D*- To replace the wild type allele of *rsbV* with the *rsbV-S56D* allele, a PCR product containing ~500 bp upstream and ~500 bp downstream of the *rsbVS56* mutation was amplified using chromosomal DNA purified from PY79 as the template and primer pair 1397/1398, digested with *BamH*I and *Sal*I, and cloned into the *BamH*I and *Sal*I sites of pMINImad2 to generate pAR227. Site directed mutagenesis was conducted on pAR227 to change the codon encoding Ser56 to a codon encoding Asp using primer pair 1401/1402 and the Quickchange II kit (Stratagene) to create pAR228. The plasmid pAR228 was introduced into PY79 and the mutant was selected as described for AR209.

Supporting Tables

Table S8: *B. subtilis* strains used in this study

**Strain**	**Genotype**
PY79	Wild type
AR209	*sspA-S47A*
AR210	*sspA-S47D*
AR187	*sspA-S47A, sspB::spc*
AR188	*sspA-S47D, sspB::spc*
AR211	*sspA-S6A,S9A,S58A*
AR212	*sspA-S6D,S9D,S58D*
AR191	*sspA-S6A,S9A,S58A, sspB::spc*
AR192	*sspA-S6D,S9D,S58D, sspB::spc*
AR227	*sspB-S45A*
AR228	*sspB-S45D*
AR229	*sspA-S47A, sspB-S45A*
AR230	*sspA-S47D, sspB-S45D*
AR231	*sspB-S45A, sspA::kan*
AR232	*sspB-S45D, sspA::kan*
AR233	*sspB S6A,S7A*
AR234	*sspB S6D,S7D*
AR235	*sspB S6A,S7A, sspA::kan*
AR236	*sspB S6D,S7D, sspA::kan*
AR186	*sspB::spc*

AR237	*sspA::kan*
AR179	*sspA::mls*
AR195	*sspA::mls, sspB::spc*
AR200	*sspA-gfp*$_{A206K}$*-spc, sspB::mls*
AR206	*sspA-S47D-gfp*$_{A206K}$*-spc, sspB::mls*
AR122	*amyE::P*$_{xyl}$*- rpsJ-spc*
AR123	*amyE::P*$_{xyl}$*-rpsJ-S32A-spc*
AR124	*amyE::P*$_{xyl}$*-rpsJ-S32D-spc*
AR168	*amyE::P*$_{xyl}$*- rpsJ-spc, rpsJ::cat*
AR169	*amyE::P*$_{xyl}$*-rpsJ-S32A-spc, rpsJ::cat*
AR185	*amyE::P*$_{xyl}$*-rpsJ-S32D-spc, rpsJ::cat*
AR223	*amyE::Pxyl- rpsJ-HA-spc-kan, rpsJ::cat*
AR224	*amyE::Pxyl-rpsJ-S32A-HA-spc-kan, rpsJ::cat*
AR225	*amyE::Pxyl-rpsJ-S32D-HA-spc-kan, rpsJ::cat*
AR121	*amyE::P*$_{hyper-spank}$*-EF-G-spc*
AR90	*amyE::P*$_{hyper-spank}$*-EF-G -Y339A-spc*
AR91	*amyE::P*$_{hyper-spank}$*-EF-G -Y339D-spc*
AR165	*amyE::P*$_{hyper-spank}$*-EF-G-spc, EF-G::cat*
AR166	*amyE::P*$_{hyper-spank}$*-EF-G-Y339A-spc, EF-G::cat*
AR167	*amyE::P*$_{hyper-spank}$*-EF-G-Y339D spc, EF-G::cat*
AR146	*amyE::P*$_{xyl}$*-EF-TU -spc*
AR147	*amyE::P*$_{xyl}$*-EF-TU-Y270*A*-spc*
AR148	*amyE::P*$_{xyl}$*-EF-TU-Y270D-spc*

AR157	*amyE::P$_{xyl}$-EF-TU-spc, EF-TU::cat*
AR158	*amyE::P$_{xyl}$-EF-TU-Y270*A*-spc, EF-TU::cat*
AR159	*amyE::P$_{xyl}$-EF-TU-Y270D-spc, EF-TU::cat*
AR213	*HPr-S46A*
AR214	*HPr-S46D*
AR129	*HPr-S46A*, c*rh:spc*
AR130	*HPr-S46D, crh:spc*
AR127	*Hpr::cat*
AR128	*Hpr::cat, crh::spc*
AR88	*crh::spc*
AR196	*hprK::spc*
SB770	*sigB::cat*
AR73	*prkC::kan*
AR102	*yabT::tet*
AR114	*yabT::tet, prkC:kan*

Table S9: Plasmids used in this study

pDR150	*amyE::PxylA-xylR (spec)*(gift from D. Rudner)
pDR111	*amyE::Phyper-spank-lacI (spec)*(gift from D. Rudner)
pMINImad2	[5]
pDG780	*B.subtilis* suicide vector with kanamycin resistance [6]
pAR100	similar to pKL147 [7] accept that the gfp gene was mutated at A206K<br>to create a monomeric variant of the protein [8]
pAR200	*sspA erm amp oriBsTs*
pAR201	*sspA-S47A erm amp oriBsTs*
pAR202	*sspA-S47D erm amp oriBsTs*
pAR203	*sspA-S6A erm amp oriBsTs*
pAR204	*sspA-S6D erm amp oriBsTs*
pAR205	*sspA-S9A erm amp oriBsTs*
pAR206	*sspA-S9D erm amp oriBsTs*
pAR207	*sspA-S6A,S9A erm amp oriBsTs*
pAR208	*sspA-S6D,S9D erm amp oriBsTs*
pAR209	*sspA-S58A erm amp oriBsTs*
pAR210	*sspA-S58D erm amp oriBsTs*

pAR211	*sspA-S6A,S9A,S58A erm amp oriBsTs*
pAR212	*sspA-S6D,S9D,S58D erm amp oriBsTs*
pAR213	*HPr erm amp oriBsTs*
pAR214	*HPr-S46A erm amp oriBsTs*
pAR215	*HPr-S46D erm amp oriBsTs*
pAR218	*amyE::P_{xyl}-rpsJ-spc*
pAR219	*amyE:: P_{xyl}-rpsJ-S32A-spc*
pAR220	*amyE:: P_{xyl}-rpsJ-S32D-spc*
pAR221	*amyE::$P_{hyper\text{-}spank}$-EF-G-spc*
pAR222	*amyE:: $P_{hyper\text{-}spank}$-EF-G Y339A-spc*
pAR223	*amyE::$P_{hyper\text{-}spank}$-EF-G Y339D-spc*
pAR224	*amyE::P_{xyl}-EF-TU-spc*
pAR225	*amyE::P_{xyl}- EF-TU-Y270A-spc*
pAR226	*amyE::P_{xyl}- EF-TU-Y270D-spc*
pAR240	*rpsJ-HA-kan*
pAR229	*sspA-gfp_{A206K}-spc*
pAR230	*sspA-S47D-gfp_{A206K}-spc*
pAR241	*sspB erm amp oriBsTs*
pAR242	*sspB-S45A erm amp oriBsTs*
pAR243	*sspB-S45D erm amp oriBsTs*
pAR244	*sspB-S6A,S7A erm amp oriBsTs*
pAR245	*sspB-S6D, S7D erm amp oriBsTs*

Table S10: Primers used in this study

Primer	Sequence 5´ -> 3´
1349	accaataaaaacagaaaaggaaaatgg
1350	acatgtattcacgaacgaaaatcgagtgtaaaatctccttttttatttagtatg
1351	attttagaaaacaataaacccttgcacaattcaatatatggctatagaggg
1352	gaaggccaatattatcaaatgtatgaa
1474	tacagagtttctgaacgtaattggc
1475	acatgtattcacgaacgaaaatcgaaagatctccccttttctttacggtt
1476	attttagaaaacaataaacccttgcagtgataaaaatgagtgactttctaaaag
1477	aaagcttgcagataacctgcaagc
1466	gtgaacttgattccggtaaactac
1467	atcacctcaaatggttcgctgggttttgatcttcaccctcttcaacttgta
1468	aagttcgctagataggggtcccgagccaaggagggaaaatatgcctgagg
1469	attgcctaaagcggcaaaatcagc
2022	gcttatggaagaagtcatccgcacaag
2023	gaacaacctgcaccattgcaagactgccagcatgccccttctt
2024	ttgatcctttttttataacaggaattcatggtgcaaactgcagagcctatg
2025	gcccaatcgagaaactgctgaatgt
2026	cctcaacccgctggtcatatcc
2027	acatgtattcacgaacgaaaatcgacaccttctttgctctattttatgcagctgt
2028	attttagaaaacaataaacccttgcaatcaaaaaggcagccgcacaag
2029	ggtcataattgaaaaggcgaatcc
2030	gcgtatcacaatgtcagcatcaaaggag

2031	attatgtcttttgcgcagtcggcggacctcctctatcatgtttgatacactatgc
2032	cattcaattttgagggttgccaggcaacgtgacccgtccgaatag
2033	ccactcttgcttctgtatgatgggc
2018	ctacctttacgtattcggcatcagctg
2424	ctgagcgagggagcagaagtctgcacttcccttggttgtact
2425	gttgaccagtgctccctgcttgtcccaaaagcggaacaagaac
2021	gaagtgaagcgtgctgatgaaatcg
1403	tagggatcccctcatgcttcagatagccgacag
1404	taggtcgactacgaccaagattcttgcttgcccg
2297	ctcgcgctaacggtgctgttggaggagag
2298	ctctcctccaacagcaccgttagcgcgag
2299	caacttctcgcgctaacggtgatgttggaggagagatcaca
2300	tgtgatctctcctccaacatcaccgttagcgcgagaagttg
1405	cacatggctaacaataacgcaggtaacagcaacaacc
1406	ggttgttgctgttacctgcgttattgttagccatgtg
1407	gaggtgagacacatggctaacaataacgatggtaacagcaacaaccttttag
1408	ctaaaaggttgttgctgttaccatcgttattgttagccatgtgtctcacctc
1829	gctaacaataacgcaggtaacgccaacaaccttttagtaccagg
1830	cctggtactaaaaggttgttggcgttacctgcgttattgttagc
1831	ggctaacaataacgatggtaacgacaacaaccttttagtaccagga
1832	tcctggtactaaaaggttgttgtcgttaccatcgttattgttagcc
1641	gagatcacaaaacgtcttgtagcttttgctcaacaaaacatgg
1642	ccatgttttgttgagcaaaagctacaagacgttttgtgatctc

1643	gagagatcacaaaacgtcttgtagattttgctcaacaaaacatgggcg
1644	cgcccatgttttgttgagcaaaatctacaagacgttttgtgatctctc
1246	tagggatccccgaatgtaccatctctcatgacaccg
1247	taggtcgacacagggttcagaagctcagggtcac
2291	acggcaaaacagttaaccttaaagctattatgggtgttatgtcttta
2292	taaagacataacacccataatagctttaaggttaactgttttgccgt
2293	atataacggcaaaacagttaaccttaaagatattatgggtgttatgtctttaggtatc
2294	gatacctaaagacataacacccataatatctttaaggttaactgttttgccgttatat
1713	aaaaaacatttaaagtaactgcagatgctggaatccatgctcgtc
1714	gacgagcatggattccagcatctgcagttactttaaatgtttttt
1715	ggcacaaaaaacatttaaagtaactgcagatgatggaatccatgctcgtc
1716	gacgagcatggattccatcatctgcagttactttaaatgttttttgtgcc
2235	taggtcgacaaaggtggtgaactactatggcaaaacaaaaaattcgtattcgtttg
2236	tagggatccttagaattaaagtttaatttcgatatcgacaccag
2287	tgaaacggcaaaacgtgctggtgccagcgtatc
2288	gatacgctggcaccagcacgttttgccgtttca
2289	gaagattgttgaaacggcaaaacgtgatggtgccagcgtatc
2290	gatacgctggcaccatcacgttttgccgtttcaacaatcttc
1775	tagaagcttaaaggtggtgaactactatggcaagagagttctccttag
2169	taggcatgcagcggcaaaatcaattattcgc
1462	gtactctggaacacttgattctggttcagccgtgaaaaactctactaaa
1463	tttagtagagtttttcacggctgaaccagaatcaagtgttccagagtac
1464	ctctggaacacttgattctggttcagacgtgaaaaactct

1465	agagtttttcacgtctgaaccagaatcaagtgttccagag
2401	atgaactttaataaaattgatttagacaattgg
2402	taaaagccagtcattaggcctatc
2233	taggtcgacaaaggtggtgaactactatggctaaagaaaaattcgaccgttcc
2234	tagggatcccacaggggtctcgtttaaaaccatact
1778	gttgaaatgttccgtaagcttcttgatgccgctgaagctggtg
1779	caccagcttcagcggcatcaagaagcttacggaacatttcaac
1780	tgaaatgttccgtaagcttcttgatgacgctgaagctgg
1781	ccagcttcagcgtcatcaagaagcttacggaacatttca
2385	gttatcgacgttgctattgagcc
2386	attatgtcttttgcgcagtcggcgccattctaaaatcctccttaagagc
2387	cattcaattttgagggttgccagtagtatggttttaaacgagacccctg
2388	gtgagctgaattctcaaattgaaacca
2403	agtagttcattgggatcagagaggatcg
2404	ccaattgtctaaatcaattttattaaagttcattattttccctccttttcgcctacatcac
2405	gaattgtcagataggcctaatgactggcttttattctaaaatatagaatgatcttaataggaggtg
2406	cttttgttttagccgtatcaagagtaa
2407	tgaagatcaaggtatctctgtctcaatg
2408	ccaattgtctaaatcaattttattaaagttcattgggtaatttctccttccttattaggaaattg
2409	gaattgtcagataggcctaatgactggcttttattgattttgccgcttaactcaagtataac
2410	gtgattgtacctggtttagcaagtac
2505	accaataaaaacagaaaaggaaaatgg
2506	ctgagcgagggagcagaa gtaaaatctccttttttatttagtatggttgg

2507	gttgaccagtgctccctg caattcaatatatggctatagaggg
2508	gaaggccaatattatcaaatgtatgaa
1397	tagggatccaaggactcgttctcggcatctcgc
1398	taggtcgacgcgaaggtgtgtacggccctagat
1399	gatgtcagctacatggacgctaccggattgggcgtttt
1400	aaaacgcccaatccggtagcgtccatgtagctgacatc
1401	agatgtcagctacatggacgataccggattgggcgttttt
1402	aaaaacgcccaatccggtatcgtccatgtagctgacatct
2704	taggagctcgttgccaactgaaaaatcagtttac
2705	tagggatccttattaagcgtaatctggaacatcgtatgggtagaattaaagtttaatttcgatatcgacacc
1941	tagcaattgaaccttttagtaccaggagctgctc
1942	tagctcgaggaattgtcctccgcccatgttttg
2420	aaaccaggtgatacgtttaaagtcg
2421	attatgtcttttgcgcagtcggc gtgtctcacctccttgtgagtata
2422	cattcaattttgagggttgccag tttacaatttcacataatggcttagg
2423	cagcagaaaacattgattttgtagcga
2892	ccaggaactagtaagtcatttgcagcgttttggttagccatgtgtaaa
2893	tttacacatggctaaccaaaacgctgcaaatgacttactagttcctgg
2894	gagctgcgccaggaactagtaagtcattatcatcgttttggttagccatgtgtaaaatctcc
2895	ggagattttacacatggctaaccaaaacgatgataatgacttactagttcctggcgcagctc
2896	ttctcctccgacagcaccgttagcgcgag
2897	ctcgcgctaacggtgctgtcggaggagaa
2898	ttctcctccgacatcaccgttagcgcgagaagttgtg
2899	cacaacttctcgcgctaacggtgatgtcggaggagaa
2900	tagggtaccgccgtaatgatttctcatttcagctgg
2901	taggtcgacgcatgttgagatctgatcagagcg
1345	aaaccaggtgatacgtttaaagtcg
1346	atcacctcaaatggttcgctgggtttgtgtctcacctccttgtgagtata

1347	aagttcgctagataggggtcccgagctttacaatttcacataatggcttagg
1348	ggtattctgtcggagtgcagca

References

1. Youngman P, Perkins JB, Losick R. Construction of a cloning site near one end of Tn917 into which foreign DNA may be inserted without affecting transposition in *Bacillus subtilis* or expression of the transposon-borne *erm* gene. Plasmid. 1984;12(1):1-9.
2. Wach A. PCR-synthesis of marker cassettes with long flanking homology regions for gene disruptions in *S. cerevisiae*. Yeast. 1996;12(3):259-65. doi:10.1002/(SICI)1097-0061(19960315)12:3<259::AID-YEA901>3.0.CO;2-C.
3. Arnaud M, Vary P, Zagorec M, Klier A, Debarbouille M, Postma P et al. Regulation of the sacPA operon of *Bacillus subtilis*: identification of phosphotransferase system components involved in SacT activity. J Bacteriol. 1992;174(10):3161-70.
4. Hanson KG, Steinhauer K, Reizer J, Hillen W, Stulke J. HPr kinase/phosphatase of *Bacillus subtilis*: expression of the gene and effects of mutations on enzyme activity, growth and carbon catabolite repression. Microbiology. 2002;148(Pt 6):1805-11.
5. Patrick JE, Kearns DB. MinJ (YvjD) is a topological determinant of cell division in *Bacillus subtilis*. Mol Microbiol. 2008;70(5):1166-79. doi:10.1111/j.1365-2958.2008.06469.x.
6. Guerout-Fleury AM, Shazand K, Frandsen N, Stragier P. Antibiotic-resistance cassettes for *Bacillus subtilis*. Gene. 1995;167(1-2):335-6.
7. Lemon KP, Grossman AD. Localization of bacterial DNA polymerase: evidence for a factory model of replication. Science. 1998;282(5393):1516-9.
8. Segev E, Rosenberg A, Mamou G, Sinai L, Ben-Yehuda S. Molecular kinetics of reviving bacterial spores. J Bacteriol. 2013;195(9):1875-82. doi:10.1128/JB.00093-13.
